# Optical spectroscopic determination of photoexcited small-polaron hopping in transition metal oxide photocatalysts

**DOI:** 10.1039/d5sc08101g

**Published:** 2026-01-05

**Authors:** Lei Tian, Michael Sachs, Lucas G. Verga, Viktoria F. Kunzelmann, Andreas Kafizas, Ian D. Sharp, Scott K. Cushing, Aron Walsh, James R. Durrant

**Affiliations:** a Department of Chemistry and Centre for Processable Electronics, Imperial College London London W12 0BZ UK l.tian@imperial.ac.uk j.durrant@imperial.ac.uk; b Department of Materials and Environmental Chemistry, Stockholm University Stockholm SE-10691 Sweden; c PULSE Institute for Ultrafast Energy Science, Stanford University Menlo Park CA 95024 USA; d Department of Materials and Centre for Processable Electronics, Imperial College London London SW7 UK; e Walter Schottky Institute, Technical University of Munich Garching 85748 Germany; f Physics Department, TUM School of Natural Sciences, Technical University of Munich Garching 85748 Germany; g Division of Chemistry and Chemical Engineering, California Institute of Technology Pasadena CA 91125 USA

## Abstract

Ultrafast small-polaron formation profoundly shapes the electronic and catalytic behaviour of transition metal oxides (TMOs). Despite its significance, spectroscopic investigations of photoexcited polaron hopping in TMOs have been scarcely explored. Here, we present the first optical spectroscopic observation of photoexcited small-polaron hopping across the first-row TMOs, using femtosecond transient absorption spectroscopy. This polaronic feature rises within 500 fs as Drude-type absorption converts to localized, polaronic absorption. Fitting with a small-polaron optical conductivity model yields polaron relaxation energies of 400–650 meV, evidencing substantial energy loss upon self-trapping. Kinetic analysis shows that oxides with open d-shells localize charge most readily: polaron formation activation barriers are low in all TMOs (0–10 meV), whereas hopping barriers remain much higher (200–350 meV). This work establishes key spectroscopic and kinetic insights, highlighting the trade-off between charge localization and mobility, as well as the critical role of polaron formation in TMOs photocatalysts.

## Abstract 2

Ultrafast small-polaron formation profoundly shapes the electronic and catalytic behaviour of transition metal oxides (TMOs).

## Graphical abstract

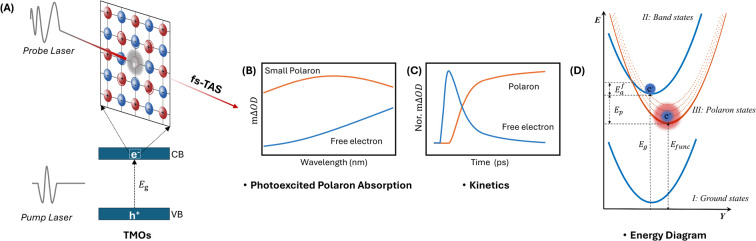

## Introduction

Polaron formation has been widely reported in transition metal oxides (TMOs).^1,2^ The significance of polaronic interactions between charge carriers and the lattice has been demonstrated in the electronic,^3^ magnetic,^4^ and optical^5,6^ properties of many TMOs. Polarons are generally classified into large polarons and small polarons, which differ primarily in the strength of the electron–lattice interaction.^7^ For small polarons, the focus of this study, the electron–lattice interaction limits the spatial extent of the polaron distortion to around a lattice constant, and the electrons (or holes) are strongly localized. The localized polaronic charge carriers migrate *via* hopping, producing a characteristic optical absorption profile that can be well-captured by a theoretical conductivity model derived from the polaron-hopping transport mechanism first proposed by Eagles,^8^ Reik^9^ and later by Bogomolov^10^ and Emin.^6^ This small polaron optical conductivity model has been experimentally validated in a range of TMOs^10–15^ using steady-state absorption spectroscopy, since polaron optical conductivity is proportional to the small-polaron hopping-induced optical absorption.^9,10,15^ This model has more recently been used to describe polaron absorption in superconductors^16^ and nonlinear optical crystals.^17^ However, application of this small polaron optical conductivity model to photoexcited polarons has been very limited to date, despite the critical role of polaronic semiconductors in solar energy harvesting.

Photoexcited small-polaron hopping-induced absorption (polaron hopping absorption) originates from a light-assisted polaronic electron transfer that occurs following polaron formation. To date, studies of polaron hopping absorption in the literature have primarily focused on polarons in the ground state of chemically doped metal oxides, without involving photoexcitation.^18^ To the best of our knowledge, aside from NiO and TiO_2_, ground-state polaron-hopping absorption spectra remain unreported for the other five TMOs studied here (BiVO_4_, Cr_2_O_3_, Mn_2_O_3_, Fe_2_O_3_, and CuO). Photoexcited polaron hopping absorption spectra have not been demonstrated or analysed for any of the seven TMOs investigated here, including NiO and TiO_2_. In the study reported herein, we make the first optical spectroscopic assignment of photoexcited small-polaron hopping for a range of TMOs with different d-shell occupancies, and show these spectra can all be fit remarkably well by the small polaron optical conductivity model. This enables the determination of key descriptors of small polarons across these oxides, including the polaron relaxation energy, phonon frequency and polaron hopping activation energy.

Recently, transient spectroscopies, particularly employing extreme ultraviolet (XUV) and X-ray detection, have been used to evaluated the polaron formation kinetics in typically ranging from tens to hundreds of femtoseconds (fs) in a range of TMOs,^19–32^ especially on two TMOs widely employed in PEC devices: TiO_2_ ^21,22,25,29,30^ and Fe_2_O_3_.^19,20,23,24,28,32^ However, a central debate persists regarding whether polaron formation involves an activation barrier: *ab initio* calculations by Yuan *et al.* report activation energies as high as 400 meV in oxides,^34,35^ while Harris and Yang argue that nuclear tunnelling eliminates any such barrier, making the process effectively barrierless.^36,37^ Our study leverages the evolution of the small polaron hopping absorption to directly monitor polaron formation, as photoexcited carriers transition from Drude-like, delocalized absorption to the characteristic localized polaron absorption. Moreover, by conducting temperature-dependent kinetic measurements, we can quantify the polaron formation activation energy, as well as correlate it with the d-shell electron occupation, thereby revealing the propensity for photoexcited charge (de)localization across the first-row TMOs.

Photoexcited small polaron formation in TMOs has significant implications for the photocatalytic (PC) and photoelectrochemical (PEC) function of these photocatalysts. For example, almost all analyses of the energetics of electrons and holes in TMOs to drive surface PC/PEC reactions employ conduction/valence band edge (CBM and VBM) energies, without consideration of the energetic loss resulting from polaronic relaxation.^38,39^ Moreover, we have recently proposed that ultrafast polaron formation may be a key driver of charge separation in open d-shell TMOs, mitigating ultrafast band-to-band recombination.^40^ Given the increasing interest in a broad range of TMOs for PC/PEC devices, it is critical to establish the generality of polaron formation and quantify the energetic loss associated with their formation across TMOs,^32,39,40^ and thus their impact on PC/PEC solar energy conversion efficiencies.

In this work, we employ femtosecond transient absorption spectroscopy (fs-TAS) to explore photoexcited small polaron formation in seven first-row TMOs (d_0_–d_9_). In the TMOs studied, we observe an ultrafast evolution of the photoinduced transient absorption from the Drude absorption of initially generated free charges to the small polaron hopping absorption of localized small polarons. Formation of localized charges is further confirmed by the observation of Jahn–Teller splitting of transient optical absorption in Fe_2_O_3_. Fitting of the photoexcited polaron hopping absorption to the small polaron optical conductivity model allows determination of the key polaron parameters for all the TMOs studied. Furthermore by studying the kinetics of polaron formation, we establish the relationship between the degree of charge localization and d-shell filling in these TMOs, with d_0_ and near-d_10_ oxides tending to delocalize charges, whereas those with partially filled d-shells favour charge localization, consistent with theoretical predictions.^32,33^ Moreover, temperature dependent kinetic measurements enable us to quantify the energetic barrier of polaron formation, revealing values that are significantly smaller than the thermal energy at room temperature. Through this work, we highlight the dynamical and energetic significance of understanding the delocalized-to-localized electronic transition in polaronic TMOs for advancing photocatalytic applications.

## Results and discussion

In this study, we focused on ultrafast transient absorption (fs-TAS) studies of thin, dense films of seven first-row TMOs with differing d-shell occupancies: d_0__BiVO_4_, d_0__TiO_2_, d_3__Cr_2_O_3_, d_4__Mn_2_O_3_, d_5__Fe_2_O_3_, d_8__NiO and d_9__CuO. Synthesis and materials characterisation details can be found in the SI. An ultraviolet pump pulse (3.5–4.1 eV) was used to generate electrons and holes in the CB and VB, respectively, and a NIR probe pulse (0.75–1.4 eV) was used to follow the evolution of the absorption of these photoexcited charges. From the steady-state absorption spectra shown in Fig. S5–S11, it is apparent that no significant ground state absorption exists in the NIR range for the TMOs studied, as such, contributions to the photoinduced transient spectra from ground state bleaching are avoided.

**Fig. 1:**
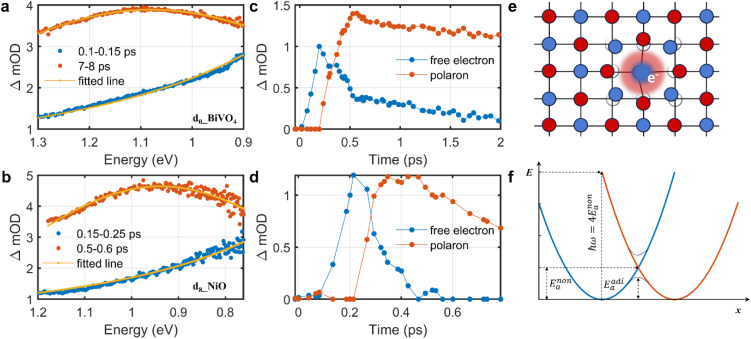
Photoinduced absorption, kinetics, and schematic description of excited charge carriers. (a and b) Averaged fs-TAS spectra from specific time windows for d_0__BiVO_4_ and d_8__NiO after excitation at 3.5 eV (355 nm, fluence of 2.5 mJ cm^−2^) and 4.1 eV (305 nm, fluence of 2.1 mJ cm^−2^), respectively. The solid orange lines are fits according to Drude model (blue data points) and small polaron optical conductivity model (brown data points) (see eqn (1)). (c and d) Kinetics of free electrons and small polarons determined for these TMOs using global fitting analyses of fs-TAS spectra as a function of time delay, using the fits to the spectra in a and b as initial inputs. (e) Schematic description of small polaron formation when an electron localizes at a cation (blue circles), illustrated for a face-centred cubic lattice (*e.g.* NiO, blue circles: cations, and red circles: anions). (f) Illustration of the small-polaron hopping model used to fit the polaron hopping absorption, plotting the electronic energy of a small polaron as function of configurational parameter *x*; ℏ*ω* is the absorbed photon energy; *E*^non^_a_ is the nonadiabatic hopping activation energy; *E*^adi^_a_ is the adiabatic hopping activation energy.

Fig. 1a–d show illustrative transient absorption spectra and corresponding kinetics for two representative TMOs BiVO_4_ and NiO. Further fs-TAS data for these two TMOs, and for all the other TMOs studied, can be found in Fig. S12–S20. It is apparent from Fig. 1a and b that we can observe distinctly different transient spectra at early time delays, 100–300 fs, and subsequently > 300 fs. In the early time range of 100–300 fs, the absorption spectra monotonically decrease with energy (rise with wavelength). At longer time delays (> circa 300 fs) the absorption spectra transform into broad absorption peaks centred circa 1 eV. A broadly similar spectral evolution was observed for all the TMOs studied, with the notable exception of Fe_2_O_3_, as discussed below.

The early time absorption spectra (100–300 fs) can be fitted well to the Drude model of free carrier absorption, Δ*A* ∝ *E*^−*α*^, where *A* and *E* denote the absorbance and photon energy, respectively, as illustrated by the orange fit lines in Fig. 1a and b (with fitted scaling exponents: *α* = 2.14 ± 0.02 for BiVO_4_, *α* = 1.90 ± 0.03 for TiO_2_, *α* = 1.95 ± 0.03 for NiO, and *α* = 1.96 ± 0.03 for CuO, see also Fig. S21, S22 and S24–S26; Fig. S23 (Cr_2_O_3_), which exhibits different behaviour, is discussed below). We note that Mn_2_O_3_ gave a lower *α* = 0.93 ± 0.02, possibly caused by mixing with localized absorption. Considering the excellent agreement between the Drude model and the early time transient absorption response, we thus conclude that the initially photoexcited charge carriers are delocalized free carriers.

After 300 fs, the transient spectra evolve into broad peaks in the NIR, as exemplified by the features at 1.1 eV and 0.95 eV in BiVO_4_ and NiO respectively (see Fig. 1a and b). Similar spectra were observed in TiO_2_, Fe_2_O_3_, Cr_2_O_3_, Mn_2_O_3_, and CuO (see Fig. S27–S34). These broad peaks are all centred at *ca.* 1 eV, with peak widths (full width at half maximum) of *ca.* 0.6 eV. For all the TMOs studied, this broad absorption peak was found to be well fitted by the small polaron optical conductivity model given by eqn (1):^6,10^1
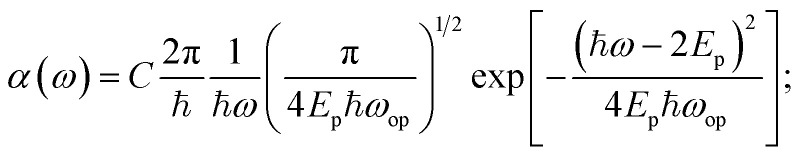
where, *α*(*ω*) is the optical absorption coefficient as a function of photon frequency (*α*(*ω*) is proportional to optical conductivity in the NIR range^9,10,15^), *E*_p_ is the polaron relaxation energy, ℏ*ω*_op_ is the energy of the lattice optical phonon coupled with the electron, ℏ is the reduced Planck constant and *C* is a scaling constant (see details in Section S7). Fits of eqn (1) to the experimental spectra are shown as the orange lines in Fig. 1a and b, and in S27–S34. The two fit parameters determined from these spectra, *E*_p_ and ℏ*ω*_op_, are listed in Table 1.

**Table 1:** Fitted parameters from eqn (1), 
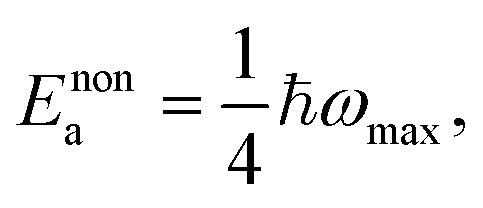
 and ref. reported parameters

		*E*_p_ (eV)	ℏ 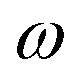 _op_ (eV)	*E* ^non^_a_ (eV)	*E*_p_ (eV)_ref_^a^	ℏω_op_ (eV) _ref_^b^	*E*_a_ (eV) _ref_^c^
d_0_	TiO_2_	0.42 ± 0.001	0.072 ± 0.003	0.21 ± 0.001	0.40	0.10	0.30; 0.13
d_0_	BiVO_4_	0.60 ± 0.001	0.11 ± 0.002	0.30 ± 0.001	0.52	0.102	0.286; 0.268
d_3_	Cr_2_O_3_	0.55 ± 0.003	0.086 ± 0.004	0.28 ± 0.003	—	0.077	0.27–0.32; 0.21–0.30
d_4_	Mn_2_O_3_	0.55 ± 0.001	0.058 ± 0.001	0.28 ± 0.001	—	—	0.30; 0.64
d_5_	Fe_2_O_3_	0.65 ± 0.001	0.033 ± 0.001^d^	0.33 ± 0.001	0.48; 0.44	0.082	0.11–0.20
d_8_	NiO	0.52 ± 0.001	0.084 ± 0.002	0.26 ± 0.001	0.52	0.71–0.80	0.20–0.25
d_9_	CuO	0.55 ± 0.002	0.097 ± 0.003	0.27 ± 0.002	—	0.077	0.272; 0.13–0.16; 0.22

The physical nature of the small polaron optical conductivity model employed in eqn (1) is illustrated in Fig. 1f.^2^ If the lattice of the metal oxide is treated as an array of molecular metal complexes, the nature of small polaron absorption is the photo-assisted electron hopping between two neighbouring ‘molecules’. This hopping process can be pictorially presented by introducing a single configurational coordinate for these two metal centres (see Fig. 1f). The quantity ℏ*ω* is the photon energy required to transfer a polaronic electron from one metal centre to the other and is associated with *E*_p_ by the relation of 
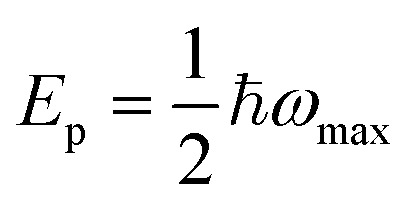
 (as indicated in eqn (1)). The electronic coupling strength between the two metal centres determines whether the electron hopping mechanism (after polaron formation) is either adiabatic or non-adiabatic, yielding hopping activation energies of *E*^adi^_a_ or *E*^non^_a_ respectively. We note 
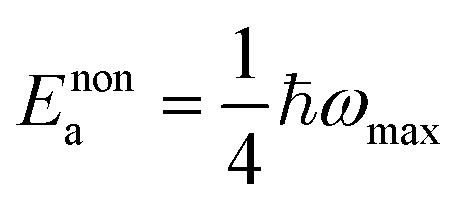
 in the non-adiabatic limit.^2^

The broad photoinduced absorption observed at delay time beyond 300 fs is well described by eqn (1), confirming its origin in small-polaron hopping. This interpretation is further supported by its close correspondence to chemically-induced small polaron absorption in doped TiO_2_ and NiO.^10,15^ Although static small polaron absorption has only been reported for TiO_2_ and NiO, for the other oxides studied, indirect evidence from their analogues also supports the polaron absorption spectra we observe. For BiVO_4_ and Mn_2_O_3_, the interpretation is supported by similar polaron bands at *ca.* 1 eV in V_2_O_5_ (^47,57^, V^5+^ as in BiVO_4_) and d_3_ manganites (^58^, Mn^3+^ as in Mn_2_O_3_). Likewise, our assignments for Cr_2_O_3_ and CuO (Fig. S29 and S34) align with previously reported electron-transfer bands at *ca.* 1 eV,^59–61^ considering the electron-transfer nature of polaron hopping absorption. Moreover, the extracted polaron relaxation energies (*E*_p_) and characteristic phonon energies (ℏ*ω*_op_) agree closely with values from prior chemical-doping or theoretical studies, as demonstrated in Table 1, despite the fundamentally different origin of our photogenerated polarons. With these unambiguous spectroscopic assignments, we can now accurately quantify polaron formation kinetics across the series.

With the assignment of our early time (<300 fs) transient spectra to free charge absorption and later time spectra to polaron hopping absorption, global analyses were performed to estimate the free charge decay and small polaron formation kinetics (see *e.g.*Fig. 1c and d). For BiVO_4_ (Fig. 1c) these analyses yielded a free electron decay half time *t*_1/2_ = 0.5 ps, and polaron rise *t*_1/2_ = 0.31 ps. Similarly for NiO, free charge decay and small polaron formation half-times were *t*_1/2_ = 0.30 ps and *t*_1/2_ = 0.27 ps respectively. These kinetics of free charge decay and polaron formation, on the 200–500 fs timescale, are in the agreement with previous reports on the sub-picosecond kinetics of polaron formation in TMOs.^19,20^

To summarize this section, we have assigned the early time (<300 fs) fs-TAS spectra to free charge carriers absorption, and the later time spectra to polaron hopping absorption in BiVO_4_, TiO_2_, Cr_2_O_3_, Mn_2_O_3_, NiO and CuO (we also note the early time spectra for Cr_2_O_3_ does not fit well to the Drude absorption model, most likely due to electron localization being initiated within our instrument response). Our analysis is based on the clear distinction between the absorption of delocalized free charge carriers *versus* localizsed small polarons, as previously emphasized by Emin^6^ and Riek.^62^ In particular, we have demonstrated that the later (>300 fs) time delay photoexcited polaron hopping absorption of these TMOs can be well described by the small polaron optical conductivity model. A delocalized-to-localized temporal evolution and the corresponding dynamics were confirmed in metal oxides of BiVO_4_, TiO_2_, Mn_2_O_3_, NiO and CuO (see Fig. S20). Key parameters *E*_p_, ℏ*ω*_op_ and *E*^non^_a_ (listed in Table 1) quantify the physical properties of the photogenerated small polarons. For all the metal oxides, the values of *E*_p_ were found to be in the range of 400–650 meV, which indicates a significant energy loss during the delocalized-to-localized transition. In addition, the values of *E*^non^_a_ determined herein were also found to be generally comparable with reported data, as indicated in Table 1, and indicative of non-adiabatic charge hopping following polaron formation as the dominant mobility mechanism in these TMOs.

**Fig. 2:**
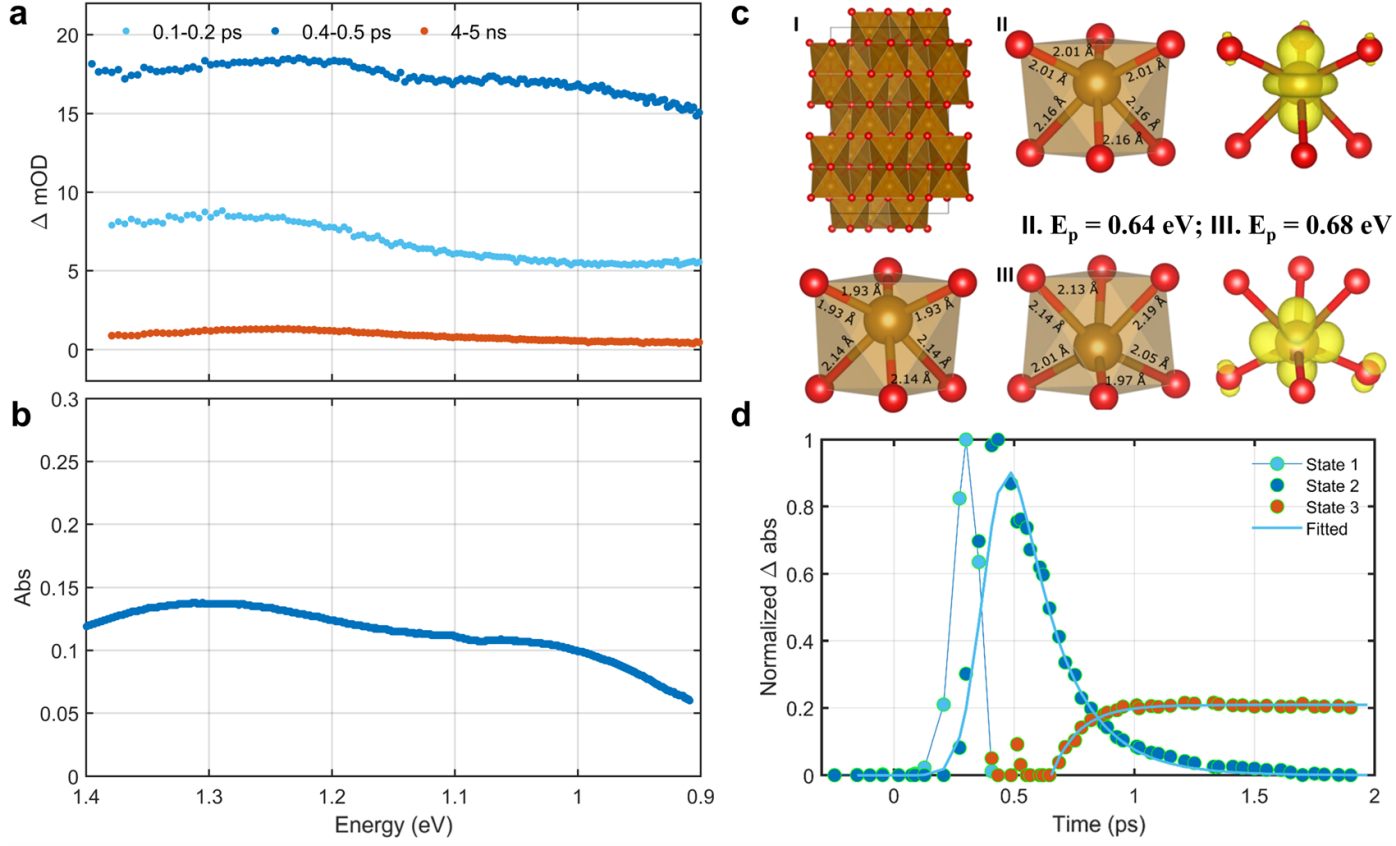
Optical absorption, kinetics, and theoretical calculations of electron polarons in Fe_2_O_3_. (a) Representative fs-TAS spectra of d_5__Fe_2_O_3_ averaged over three time-delay windows. (b) Ground state NIR absorption spectrum of a saturated aqueous FeCl_2_ solution. (c) (I). DFT calculations of the pristine crystal structure (top left) of Fe_2_O_3_ with an electron delocalized in its supercell (bottom left); an electron localized in the Fe_2_O_3_ supercell to simulate small polaron formation by (II): bond distortion method (middle), and (III): ShakeNBreak distortion method (right). (d) Global fitting of the fs-TAS spectra for d_5__Fe_2_O_3_ using the three representative spectra in a, labelled as states 1, 2 and 3 based on their sequential time evolution order. The kinetic traces (light blue lines) of states 2 and 3 were fitted with a single exponential model.

Distinct, complementary evidence for ultrafast charge localization in Fe_2_O_3_ emerges from our fs-TAS data. At 0.4–0.5 ps, Fe_2_O_3_ displays two absorption bands at 1.03 and 1.24 eV, unlike the single peak seen in other TMOs. Remarkably, these features mirror the 1.03 and 1.31 eV d–d transitions of octahedral [Fe(H_2_O)_6_]^2+^ complexes (see Fig. 2b), implying that photoexcitation in Fe_2_O_3_ transiently generates Fe^2+^ sites by polaronic electron localization. We therefore assign the split peaks to internal d–d transitions of photogenerated Fe^2+^, dynamically stabilized by a Jahn–Teller distortion.^63,64^ This agreement confirms the ultrafast formation of small electron polarons in Fe_2_O_3_. Early-time (100–300 fs) spectra deviate from Drude-like behaviour (see Fig. 2a), indicating that electron localization is triggered within our ∼100 fs instrument response.^65,66^ Beyond 0.65 ps, a single band at 1.24 eV (see Fig. 2a and S16b) emerges, identical to the Fe^3+^ → Fe^2+^ intervalence transition in Fe-doped sapphires,^67,68^ and is thus assigned to polaron hopping absorption. Small polaron optical conductivity model fitting (eqn (1)) yields a relaxation energy, *E*_p_ = 0.62 eV, and a non-adiabatic hopping barrier, *E*_a_ = 0.31 eV (Table 1), in excellent agreement with *ab initio* hopping calculations (∼0.34 eV).^52^ The fitted phonon energy of 33 meV differs from the ∼80 meV value generally reported in the literature. Recently, Knowles *et al.* showed that two phonon modes, at 31 and 81 meV, are involved in small polaron formation in Fe_2_O_3_, arising from an electron localized on two adjacent Fe atoms and associated with two types of lattice distortion. Our phonon energy of 33 meV agrees very well with the lower mode identified by Knowles *et al.*, suggesting that polaron hopping in Fe_2_O_3_ is dominated by this lower-mode channel for *t* > 0.65 ps.^44^

To validate our assignment of the 1.24 eV feature to small-polaron hopping in Fe_2_O_3_, we performed spin-polarized DFT + U simulations (see Section S5 and Table S2). In our calculations, the polaron was modelled as a single-particle excess charge, consistent with the formation of locally excess Fe^2+^ sites upon polaronic electron localization. Excitation-energy-dependent measurements on Fe_2_O_3_ further indicate that polaron hopping absorption and *E*_p_ are independent of the initial electronic state of the hot electrons (see Fig. S31b), thereby supporting the relaxed-state model employed here. Fig. 2c (I) shows the pristine Fe_2_O_3_ lattice with an extra electron (top left) and the corresponding FeO_6_ octahedron (bottom left). Upon polaron formation, localizing the excess electron on one Fe site, both the bond distortion and ShakeNBreak methods yield very similar local structures (Fig. 2c II and III, center) and electron-density maps (right). Crucially, both approaches predict comparable polaron relaxation energies (*E*_p_ = 0.64 eV for bond distortion and 0.68 eV for ShakeNBreak), in excellent agreement with our experimental absorption peak at 1.24 eV (where 
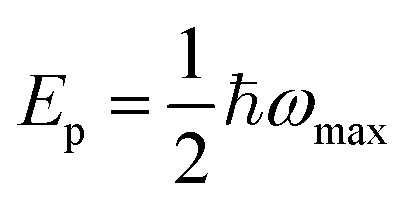
), thereby confirming the small polaron origin of this spectral feature.

**Fig. 3:**
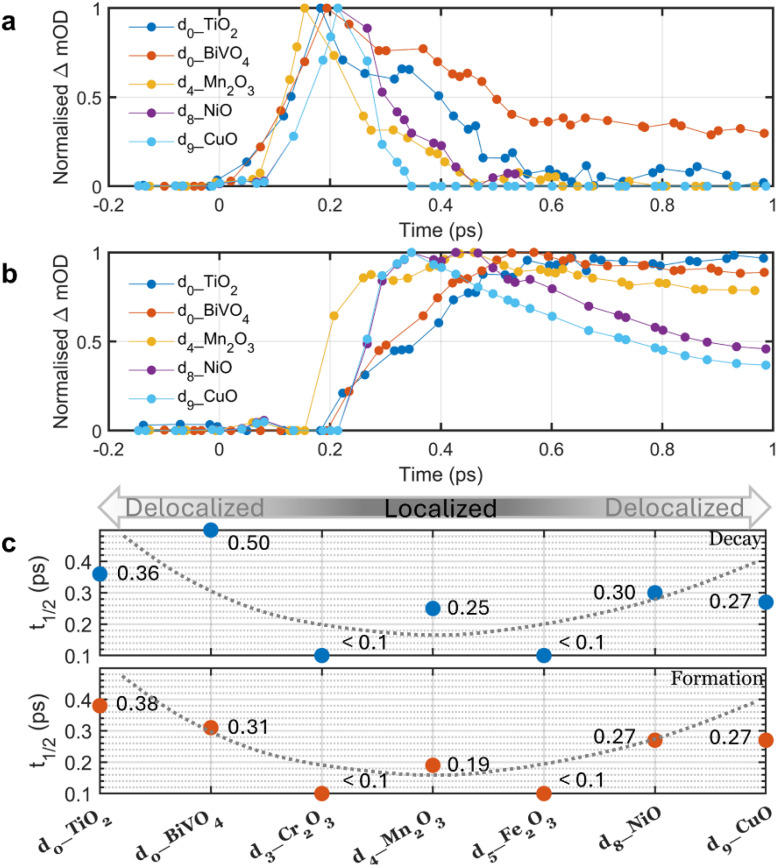
Comparison of free charge carrier decay and small polaron formation kinetics in first-row TMOs. (a) Free charge carrier decay. (b) Small polaron formation. Both a and b were extracted from global analyses. (c) Summary of the kinetic halftimes for free charge carrier decay and polaron formation. For d_3__Cr_2_O_3_ and d_5__Fe_2_O_3_, the instrument response time of *ca.* 100 fs was used as the upper limit to the kinetic response; the overlaid grey dashed curves were drawn as a guide to the eye.

Comparison of the excited-state kinetics across the full d_0_–d_9_ series can reveal the impact of d-shell occupancy on charge localization. As shown in Fig. 3a and b, the d_0_ oxides (BiVO_4_, TiO_2_) with empty d-shell exhibit the slowest localization, whereas the nearly filled d_8_–d_9_ oxides (NiO, CuO) are substantially faster. For Cr_2_O_3_ (d_3_) and Fe_2_O_3_ (d_5_), localization occurs faster than our instrument resolution; we therefore assign an upper limit of 100 fs (see Fig. S23 and 2a). Fig. 3c summarizes these trends: oxides with empty or nearly closed d-shells localize charge carriers more slowly, while those with half-filled to nearly full shells localize most rapidly (*t*_1/2_[d_0_] > *t*_1/2_[d_3_–d_5_] < *t*_1/2_[d_8_–d_9_]). The root cause of this behaviour is the strength of cation–cation coupling: for metal oxides with empty and nearly closed d-shells, cation–cation interactions are generally larger than oxides with open d-shells, resulting in wider bands and greater charge delocalization. In contrast, metal oxides with open d-shells typically exhibit weaker cation–cation interactions, resulting in narrower bands and promoting electron localization.^33^ We have recently proposed that polaron formation is in kinetic competition with ultrafast charge trapping and, for open d-shell metal oxides, ultrafast relaxation through ligand field states.^40^ Here, using a polaron formation perspective, we experimentally demonstrated the relationship between d-shell filling and the propensity for charge (de)localization in TMOs, revealing that those with open d-shells possess a greater propensity for charge localization, in the agreement with our previous work,^40^ as well as the theoretical studies.^32,33^

**Fig. 4:**
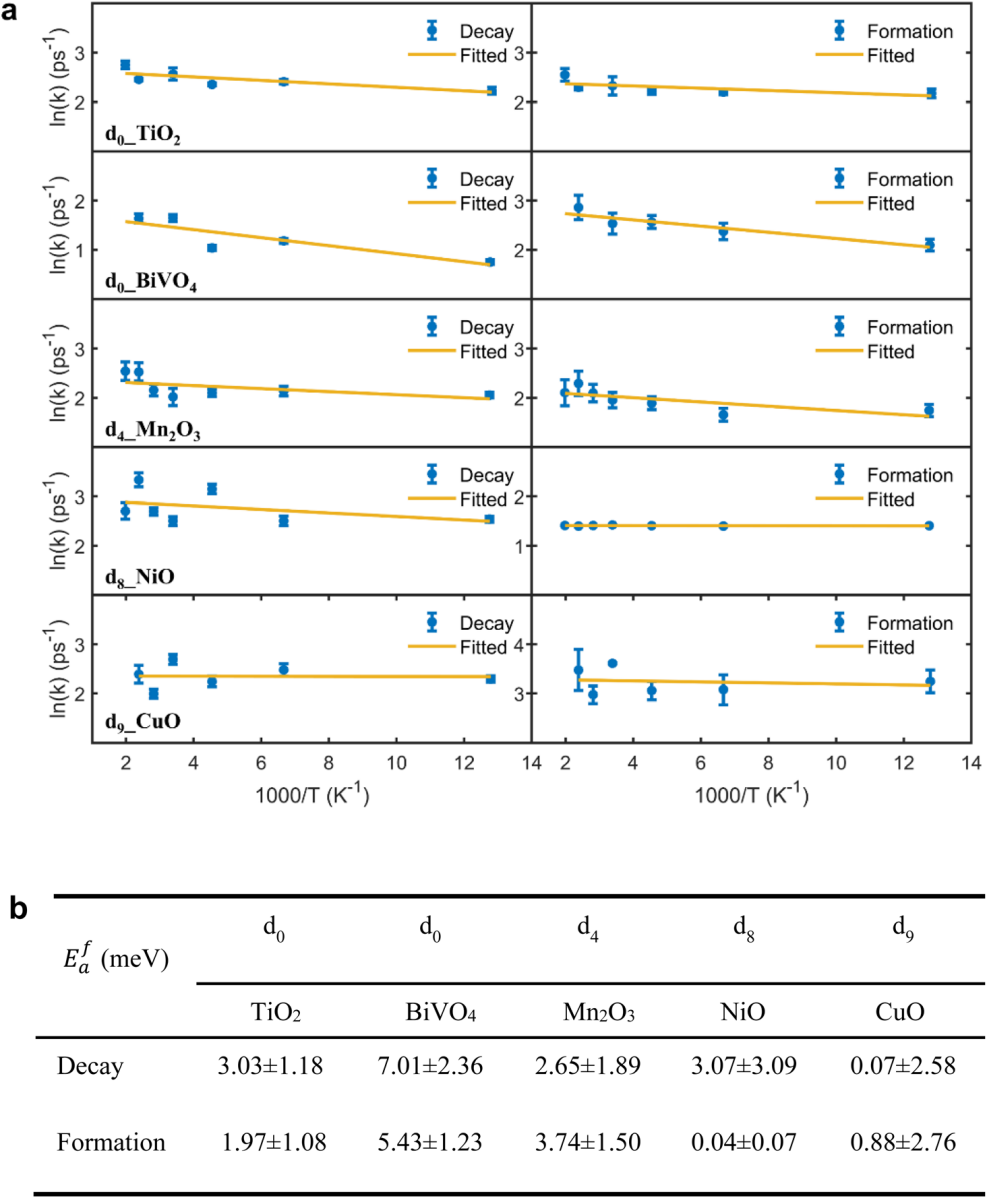
Temperature-dependent kinetics of free charge carrier localization and polaron formation. (a) Arrhenius model fitting of the temperature dependent kinetics of free charge carrier decay (left column) and polaron formation (right column). (b) Table summarizing the corresponding activation energy from the fittings of temperature-dependent carrier localization kinetics. For NiO, we obtained polaron-formation rate constants from decay half-times because single-exponential fitting gave poor fitting.

As a further extension of these kinetic studies, temperature-dependent measurements enable us to address the long-debated question of whether small polaron formation involves an activation energy (*E*^f^_a_).^34,35^ To quantify *E*^f^_a_, we collected fs-TAS data as a function of temperature for TMOs in which the absorption feature of free charge carriers and small polarons can be unambiguously distinguished (see Fig. S35–S88). As shown in Fig. 4, clear but weak Arrhenius temperature dependencies were observed in d_0__TiO_2_, d_0__BiVO_4_, and d_4__Mn_2_O_3_, yielding *E*^f^_a_ values of 2–7 meV for both free charge decay and small polaron formation. In contrast, d_8__NiO and d_9__CuO showed no detectable thermal activation within our experimental uncertainty, indicating barrierless polaron formation (*E*^f^_a_ ≈ 0; see Fig. 4b). Although theory has predicted the existence of such thermal barriers,^69,70^ to our knowledge no direct experimental verification has been reported.^34,35^ On the other hand, previous observations of non-Arrhenius temperature dependence (*E*^f^_a_ in negative value from fitting) were attributed to nuclear tunnelling processes, implying barrierless electron self-trapping.^36,37^

**Fig. 5:**
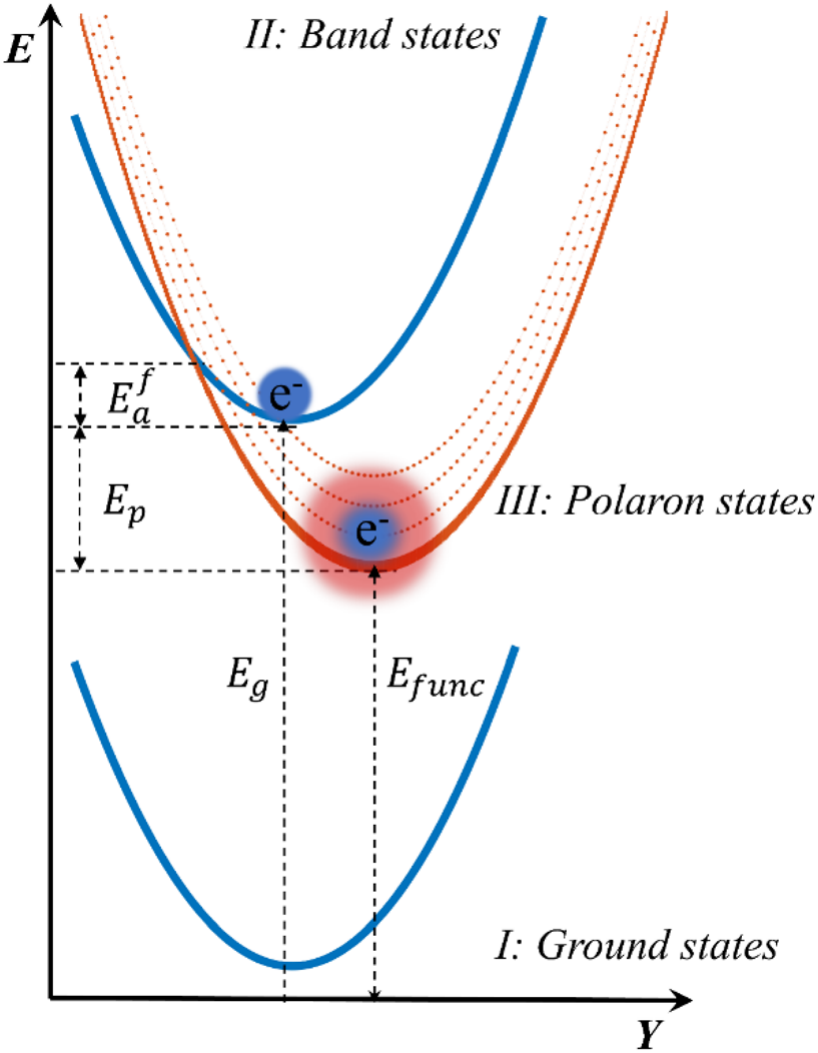
Electronic potential energy diagram of small polaron formation. Schematic illustration of the electronic potential energy as a function of configurational parameter *Y*, comparing the ground state surface with those of excited band-like and polaronic states. *E*^f^_a_ is the polaron formation activation energy, and excited multi-phonon modes are indicated by dashed lines in II: Polaron states. *E*_g_ is the optical bandgap, *E*_func_ is the functional energy level (*E*_func_ = *E*_g_ − *E*_p_) corresponding to the enthalpy stored in the photogenerated polarons. While this diagram represents localization of excited electrons, similar energy surfaces can be constructed for small hole polarons.

Herein, we experimentally demonstrate that a small thermal barrier (*E*^f^_a_ up to 7.01 ± 2.36 meV) is present for small polaron formation at least in d_0_ TMOs. The small polaron formation can thus be viewed as a non-equilibrium electron-transfer process, with the observed small barriers arising from coupling to high-frequency, multi-phonon modes, as described by the Bixon–Jortner electron-transfer model^71^ (see Fig. 5). Although polaron formation barrier variations across TMOs are subtle, d_0_ oxides consistently exhibit higher barriers, reflecting their slower polaron formation kinetics and extended lifetimes (see Fig. 3).^40^

For PC/PEC applications of semiconducting TMOs, polaron formation fundamentally redefines the functional bandgap, *E*_func_, which sets the enthalpy available in photogenerated carriers to drive interfacial redox reactions. Traditionally, the bandgap is taken from absorption or photoluminescence measurements as the optical gap, *E*_g_, which is assumed to equal, *i.e. E*_g_ = *E*_func_.^38^ However, in every TMO we studied, ultrafast polaron relaxation incurs an energy loss *E*_p_ of 400–650 meV, occurring in < 1 ps, faster than almost all interfacial reactions. In n-type semiconductors such as TiO_2_, Fe_2_O_3_ and BiVO_4_, this loss is dominated by electron self-trapping, effectively lowering the electronic energy to *E*_func_ (*E*_func_ = *E*_g_ − *E*_p_), *i.e.* 400–650 meV below the CBM, as illustrated in Fig. 5.

Polaron-formation energy loss has profound implications for the reactivity of photoexcited carriers. The loss we observe (400–650 meV) is comparable to the energy dissipated when electrons trap at defects, such as oxygen vacancies. However, whereas defect-related losses can be mitigated by synthetic control of the vacancy density or *via* defects passivation afterward, polaron relaxation represents a more intrinsic limit the solar conversion efficiency of TMOs. We note that the impact of polaron formation is not all negative; we have recently argued that polaron localization in TMOs can be a key driver of charge separation, mitigating ultrafast band to band recombination mediated by defect or ligand field states.^40^

## Conclusion

In summary, our study establishes an important spectroscopic and kinetic insights into photoexcited small polaron behaviour in TMOs, emphasizing their critical role in PC/PEC applications. This study is based on the first optical spectroscopic observation of photoexcited small-polaron hopping across the series of first-row TMOs. Polaron formation in TMOs is ultrafast (<500 fs), inevitable, and occurs with minimal activation barriers (0–10 meV), dramatically outpacing the timescales of typical interfacial PC/PEC reactions. Consequently, polaronic effects must be explicitly included in mechanistic models. Once formed, polarons undergo thermally activated hopping with large activation energies (200–350 meV), significantly limiting charge transport. The substantial polaron relaxation energy (400–650 meV) reduces the functional electronic bandgap, decreasing the driving force for interfacial electron transfer but also suppressing rapid recombination, thereby prolonging carrier lifetimes. Finally, we highlight that TMOs with d_0_ and near-d_10_ electron configurations exhibit slower polaron formation kinetics, higher energy barriers, and longer lifetimes, indicating that d-shell engineering is a promising strategy to enhance PC/PEC performance.

## Author contributions

LT conceived the project. MS was involved in the project discussion all along, prepared the Cr_2_O_3_, Mn_2_O_3_, Fe_2_O_3_ and NiO films, and wrote the global analyses code. LGV did the theoretical calculations of Fe_2_O_3_. AW supervised the theoretical calculations and made the first suggestion to calculate the small polaron formation activation energy. AK prepared the CuO film and did the XRD pattern fitting and interpretation. VFK prepared the BiVO_4_ film and collected XRD raw data of BiVO_4_, and IDS made comments for this manuscript. SC contributed to understanding of the underlying concepts and data interpretation. LT did all the rest of the experiments and data analysis. JRD supervised the whole project and proposed the outline of the manuscript framework. LT and JRD wrote this manuscript with input from all other co-authors.

## Conflicts of interest

The authors declare no competing interests.

## Supplementary Material

SC-OLF-D5SC08101G-s001

## Data Availability

The data supporting this article have been included as part of the supplementary information (SI): materials preparation, characterization methods (XRD, UV-vis-NIR absorption, fs-TAS, and temperature dependent fs-TAS), DFT + U calculation, and fitting details (Drude and small polaron absorption model fitting, and *E*^f^_a_ for polaron formation). See DOI: https://doi.org/10.1039/d5sc08101g.
