# Supplementary material for: Optical spectroscopic determination of photoexcited small-polaron hopping in transition metal oxide photocatalysts
Source: Chem Sci. 2026 Jan 5;17(8):4203–12. doi: 10.1039/d5sc08101g (PMC12784217; doi:10.1039/d5sc08101g)
Supplement: SC-017-D5SC08101G-s001 [file SC-017-D5SC08101G-s001.pdf]

Supplementary Information (SI) for Chemical Science.
This journal is © The Royal Society of Chemistry 2026

Supplementary Information for

Optical Spectroscopic Determination of Photoexcited Small-Polaron Hopping in Transition Metal Oxide Photocatalysts

Lei Tian[a,b]*, Michael Sachs[a,c], Lucas G. Verga[d], Viktoria F. Kunzelmann[e,f], Andreas Kafizas[a], Ian D. Sharp[e,f], Scott K. Cushing[g], Aron Walsh[d], James R. Durrant[a]*

[a]Department of Chemistry and Centre for Processable Electronics, Imperial College London, London W12 0BZ, U.K.

[b]Department of Materials and Environmental Chemistry, Stockholm University, Stockholm SE-10691, Sweden.

[c]PULSE Institute for Ultrafast Energy Science, Stanford University, Menlo Park, CA 95024, USA.

[d]Department of Materials and Centre for Processable Electronics, Imperial College London, London SW7, U.K.

[e]Walter Schottky Institute, Technical University of Munich, Garching, 85748, Germany.

[f]Physics Department, TUM School of Natural Sciences, Technical University of Munich, Garching, 85748, Germany.

[g]Division of Chemistry and Chemical Engineering, California Institute of Technology, Pasadena, CA. 91125, USA.

*Corresponding author. Email: l.tian@imperial.ac.uk; j.durrant@imperial.ac.uk

1. Materials preparation: The synthesis of all the metal oxides used in our study have been reported previously, with the exception for Mn_2O_3 and CuO. The synthesis of $BiVO_4$ films was reported in ref.[1]. Single crystal TiO_2 (100) was purchased from MTI Corporation (we note a single crystal sample was used for TiO_2), reported in ref.[2]. The syntheses of Cr_2O_3, Fe_2O_3, and NiO films were reported in ref.[3]. The syntheses of Mn_2O_3 and CuO films used in this study are described below.

Preparation of Mn_2O_3: Mn_2O_3 thin films were synthesised by adapting our previously used Fe_2O_3 sol-gel synthesis procedure.[3] $MnCl_2 \cdot 4H_2O$ (0.66 g, 3.33 mmol) and citric acid (0.64 g, 3.33 mmol) were dissolved in ethanol (10 mL). The resulting solution was stirred in a closed round bottom flask at 60 °C for 6-7 h. After this time, DMF 20 μL was added as a drying control reagent and the solution was stirred for additional 30 min. The solution was then spin coated onto quartz glass substrates (5000 rpm, 50 s) and the deposited films were annealed in air (20 min at 120 °C). A second layer was then spin coated on top of the first one (5000 rpm, 50 s) and the films were annealed again (20 min at 120 °C). Finally, the films were annealed at 550 °C (ramp: 10 min to 80 °C, 20 min at 80 °C, 1 h to 550 °C, 4 h at 550 °C) to complete the conversion to Mn_2O_3 (confirmed by structural analyses in Section S2). The films were then allowed to cool to room temperature within the oven.

Preparation of CuO: CuO was synthesis by an aerosol-assisted chemical vapour deposition method based on literature reported pathway[4, 5]. Details on the set-up used are published elsewhere[6]. Copper (II) nitrate hemipentahydrate (0.1 M) in methanol (40 mL) was aerosolised an ultrasonic humidifier (2 MHz, Liquifog, Johnson Matthey) and carried over the heated quartz substrate held at 350 °C using compressed air at a flow rate of 2 litres per minute (MFC, Brooks) over a period of ~20 min until the solution was fully transferred. The sample was then annealed in air at 500 °C for 12 hrs to ensure full conversion of any potential Cu or Cu_2O impurities into the CuO (confirmed by structural analyses in Section S2).

2. Materials structural characterisation: Apart from Mn_2O_3 and CuO, the structural characterisation of all samples used in this study have been reported previously[1-3, 7]. The collected X-ray diffraction (XRD) data of $BiVO_4$, TiO_2, Mn_2O_3, and CuO were further fitted based on Le

Bail model with using standard parameters from the Physical Sciences Data-Science database. All these XRD parameters were summarised in Table S1, and the XRD parameters for the rest of TMOs (Cr_2O_3, Fe_2O_3, and NiO) used in this study can be found in ref.[3]. All materials are in dense thin films with thicknesses ranging from 30 to 130 nm, except for bulk single crystal TiO_2 (0.5 mm in thickness).

3. Femtosecond transition absorption spectroscopy (fs-TAS): The fs-TAS setup used in this work is based on a regeneratively amplified Ti:sapphire laser (Solstice, Spectra-Physics) that is pumped by an intracavity-doubled, Q-switched, diode-pumped Nd:YLF laser (Empower systems, Spectra-Physics) and seeded by a diode-pumped, mode-locked Ti:sapphire laser (Mai Tai system, Spectra-Physics). The output from the regenerative laser comprises 800 nm laser pulses with a temporal width of 92 fs at a 1 kHz repetition rate. The 800 nm beam is subsequently divided into two parts, which are used to generate pump and probe pulses. The pump portion is directed to an optical parametric amplifier (TOPAS Prime, Light Conversion) and a frequency mixer (NirUVis, Light Conversion), which allows generation of pulses with specific wavelengths and can be tuned from 290 nm to the NIR region. The pump pulse is then directed through a depolarizer and is focused on the sample as the excitation light source.

The probe portion of the 800 nm pulse is first directed to a delay stage that allows pump-probe delay times of ~ 6 ns. After the delay stage, the probe pulse is focussed into an yttrium aluminium garnet (YAG) crystal in which a NIR continuum (850–1650 nm) is generated via self-phase modulation. The continuum probe pulse is split into two parts using a semi-transparent mirror, with one portion used to capture the signal of interest and the other to function as a reference that mitigates the effects of beam fluctuations, thereby enhancing the signal-to-noise ratio. The 'signal' and 'reference' probe pulses are collected using separate multichannel spectrometers (Si or InGaAs sensors) transmitted through optical fibres.

An automated femtosecond transient absorption spectrometer (Helios, Ultrafast Systems) is used for the pump-probe measurement and data collection. Prior to data collection, temporal and spatial overlap between the pump and 'signal' probe pulses is achieved. The transmitted probe pulses with and without pump pulse are measured using an optical chopper rotating at 500 Hz. The transient

absorption signal ΔA is calculated according to $\Delta A = -\log(\frac{I_w}{I_{wo}})$, where I_w is the transmitted probe pulse intensity with pump and I_{wo} is the one without pump. The energies of the pump pulse were measured using an energy meter (VEGA P/N 7Z01560, OPHIR Photonics), and the laser fluences of 2.1-2.9 mJ cm^{-2} were estimated with a 500 μm diameter aperture at room temperature in air.

4. Temperature dependent fs-TAS characterization: An optical cryostat (Oxford Instruments OptistatDN-V) was used to control the temperatures between 78-505 K. The cryostat was inserted at the sample position in fs-TAS setup to perform temperature dependent fs-TAS. During the measurement, the temperatures were changed randomly. After each temperature setpoint, the system was allowed to stabilize for 20 min before data collection to ensure a uniform temperature inside the cryostat. All the samples were found to be stable over the measured temperature range.

5. Density functional theory (DFT+U) calculations: Total energy calculations were performed using spin-polarised density functional theory (DFT+U) as implemented in the Vienna *ab-initio* Simulation Package (VASP) [8, 9] using the rotationally invariant DFT+U formalism proposed by Dudarev et al[10]. We employed the projector-augmented wave (PAW) method and the Perdew, Burke and Ernzerhof exchange-correlation functional together with the Hubbard U correction of U=4.0 eV for Fe atoms, which was determined after a series of calculations. In all the calculations, we employed a 2x2x1 supercell with 120 atoms (the charge of the simulation cell was set using the NELECT tag within VASP, which includes a homogeneous background charge for charged simulation cells), a plane wave kinetic energy cutoff of 500 eV, and 3x3x2 *k*-points mesh for the Brillouin zone integration. The convergence criteria were set to 10^{-5} eV for the electronic self-consistent iteration and 0.01 eV/Å for the atomic forces on all atoms during ionic relaxations.

The polaron bonding energy E_p was calculated as the total energy difference between two models of α-Fe_2O_3 containing an additional electron that could be either delocalised or localised [11]. For the delocalised state, the pristine bulk α-Fe_2O_3 structure was used, and the total energy of the system was calculated by adding an extra electron to the supercell. For the localised state, two different strategies were employed to model polaron formation. The first strategy relied on the bond distortion method [12], in which Fe-O bonds around a specific Fe atom were symmetrically elongated and the structure was used as an initial configuration for geometry optimization

calculations. For the second strategy, we applied the ShakeNBreak python package [13, 14] to generate ten initial configurations that contained chemically guided bond distortions and rattling around the same specific Fe atom. The first method is an efficient way to guide the formation of polarons during electronic structure calculations. The second method follows a general and automatic strategy to build distorted structures for point defects in solids, which was shown to enable the identification of low energy defect structures for different materials. In both strategies, the magnetic moment of a specific Fe atom was changed to accommodate the additional electron.

For the bond distortion method, the FeO_6 that contained the excess electron retained similar characteristics to the pristine one, with three shorter and three longer Fe-O bonds. The shorter Fe-O were elongated from 1.93 Å in the pristine and delocalised solution to 2.01 Å following localization, while the longer bonds increased from 2.14 to 2.16 Å, Fig. 2c (II). Meanwhile, upon geometry optimization with an excess electron, all ten initial structures generated via the ShakeNBreak method yielded α-Fe_2O_3 structures with the same structural characteristics and energies, Fig. 2c (III). In this case, the FeO_6 with the excess electron became more asymmetric compared to the pristine α-Fe_2O_3, with Fe-O ranging from 1.97 Å to 2.19 Å. The structure obtained with the ShakeNBreak method is slightly favoured to the one obtained from the bond distortion method, showing that the method can be useful to find other solutions for localized excess electrons that can be energetically favourable, or at least comparable, to the ones found via standard modelling approaches.

The Hubbard U correction of U=4.0 eV for Fe atoms was determined after a series of calculations and is similar to the U=4.3 eV value used for bulk α-Fe_2O_3 in the literature[15, 16]. Table S1 summarizes the results obtained with different values of U, showing that the usage of U=4.0 eV results in bandgap values close to the experimental bandgap of nearly 2.2 eV[17-19]. For reference, we also provide the results obtained with HSE06 functional[20] for pristine α-Fe_2O_3, the results of magnetic moment for the Fe atom with and without the polaron formation using the bond distortion method, the polaron binding energies with different values of U and the polaron binding energy calculated with the HSE06 functional from the literature[11].

6. Steady-state Uv-Vis-NIR absorption spectroscopy characterizations

The steady state Uv-Vis-NIR absorption spectra were performed in Cary 7000 Universal Measurement Spectrophotometer (UMS). Note the step at ~ 850 nm in some spectra is an instrument artefact.

7. Small polaron absorption analysis.

In Equation 1, $C = \frac{2Ze^2a^2n_0}{c} J^2$, in which J is the electronic coupling matrix element; Z is the number of nearest neighbours for polaron hopping; e is the elementary charge; a is the lattice constant; n_0 is the small polaron density; c is the speed of light in vacuum. We used C as a fitting constant without considering the details parameters included.

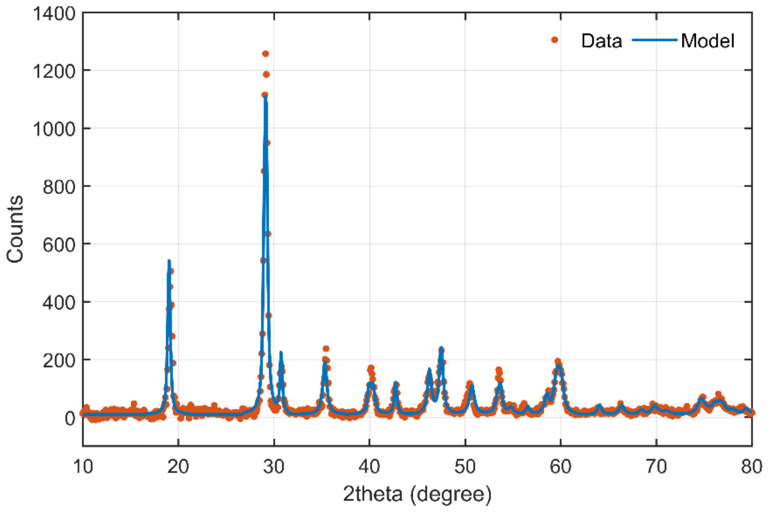

Fig. S1. XRD pattern of $BiVO_4$ sample compared to the reference data [21]. The instrumentation of raw data collection can be found in ref.[1].

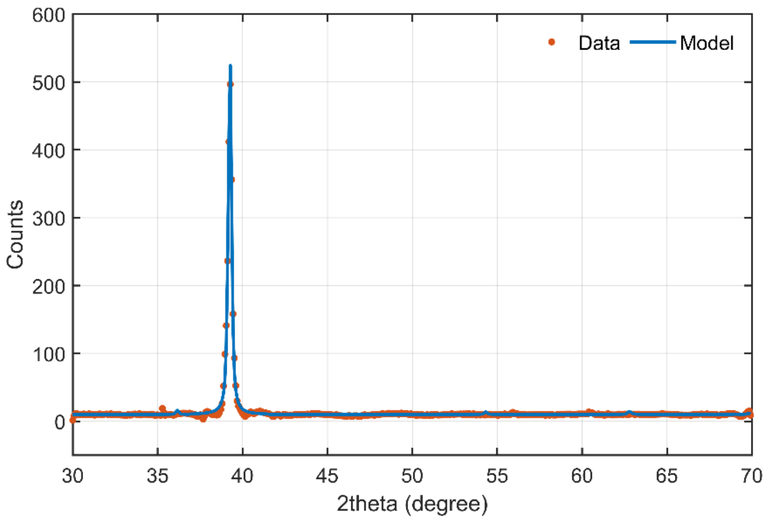

Fig. S2. XRD pattern of TiO_2 sample compared to the reference data [22]**.** The raw data were collected in $\theta - 2\theta$ mode with X-ray beam of 8.040 keV (Cu Kα), performed in a Bruker Focus D8 diffractometer.

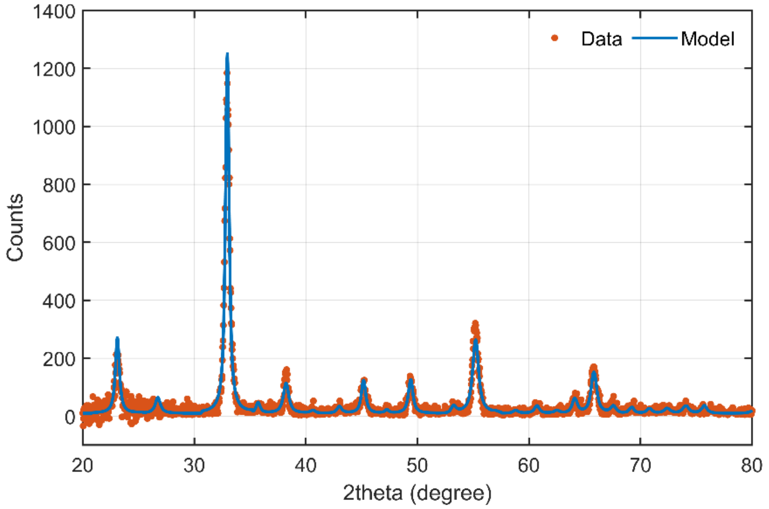

Fig. S3. XRD pattern of Mn_2O_3 sample compared to the reference data [23]. The raw data were collected in grazing incidence mode with X-ray beam of 8.040 keV (Cu Kα) in an incident angle of 0.3°, performed in a PANalytical Empyrean diffractometer.

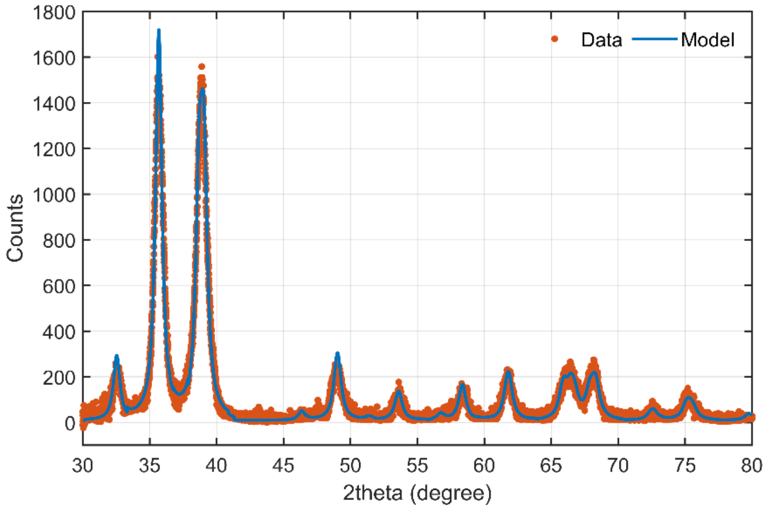

Fig. S4. XRD pattern of CuO sample compared to the reference data [24]**.** The raw data were collected in grazing incidence mode with X-ray beam of 8.040 keV (Cu Kα) in an incident angle of 0.3°, performed in a PANalytical Empyrean diffractometer.

Table S1. XRD parameters summary. Part of TMOs parameters was summarized here, and the rest can be found in ref. [3].

	Space group	Crystal system	lattice parameter (Å)					
			a		b		c	
			value	error	value	error	value	error
$BiVO_4$ standard	$I4_1/a$ (88)	Tetragonal	5.1050	0.0010	5.1050	0.0010	11.5770	0.0010
$BiVO_4$ sample			5.0871	0.0008	5.0871	0.0008	11.6189	0.0026
TiO_2 standard	$P4_2/mnm$ (136)	Tetragonal	4.5941	0.0001	4.5941	0.0001	2.9589	0.0001
TiO_2 sample			4.5920	0.0002	4.5920	0.0002	2.9576	0.0012
Mn_2O_3 standard	Ia-3 (206)	Cubic	9.4146	0.0001	9.4146	0.0001	9.4146	0.0001
Mn_2O_3 sample			9.4130	0.0010	9.4130	0.0010	9.4130	0.0010
CuO standard	C12/c1 (15)	Monoclinic	4.6837	0.0005	3.4226	0.0005	5.1288	0.0006
CuO sample			4.6752	0.0005	3.4234	0.0004	5.1226	0.0006

	unit cell angles (°)				volume ($Å^3$)			Average crystal size		Fit
	α	β		γ						
	value	value	error	value	value	error	change (%)	LX	τ (nm)	wRp
$BiVO_4$ standard	90	90	-	90	301.71	-	-	-	-	-
$BiVO_4$ sample	90	90	-	90	300.678	0.095	-0.34	23.3	34.1	0.2330
TiO_2 standard	90	90	-	90	62.450	-	-	-	-	-
TiO_2 sample	90	90	-	90	62.367	0.025	-0.13	14.9	53.4	0.0934
Mn_2O_3 standard	90	90	-	90	834.460	-	-	-	-	-
Mn_2O_3 sample	90	90	-	90	834.047	0.274	-0.05	51.8	15.3	0.5377
CuO standard	90	99.54	0.01	90	81.08	-	-	-	-	-
CuO sample	90	99.18	0.01	90	80.938	0.01	-0.18	49.7	16.0	0.3636

Standard samples referred to $BiVO_4$ [21], TiO_2 [22], Mn_2O_3 [23], and CuO [24]. The crystal forms of TiO_2 and $BiVO_4$ are rutile and tetragonal scheelite, respectively.

Table S2: Calculations to determine the Hubbard U correction of U=4.0 eV for Fe atoms. The value of U=4.0 used in this work is close to the U=4.3 eV value used in the literature and can reproduce the bandgap of nearly 2.2 eV[17-19] experimentally determined for α-Fe_2O_3.

	Pristine α-Fe_2O_3		Polaron Bond Distortion Method for α-Fe_2O_3	
	Bandgap (eV)	Fe (μ_B)	Fe (μ_B)	E_p (eV)
PBE	0.62	3.56	-	
PBE+3.5	2.00	4.06	3.56	0.46
PBE+4.0	2.17	4.08	3.56	0.64
PBE+4.3	2.27	4.12	3.56	0.74
PBE+4.5	2.34	4.13	3.56	0.80
PBE+5.0	2.51	4.16	3.57	0.94
PBE+5.5	2.67	4.19	3.58	1.26
HSE06	3.49	4.16	-	0.49[15]

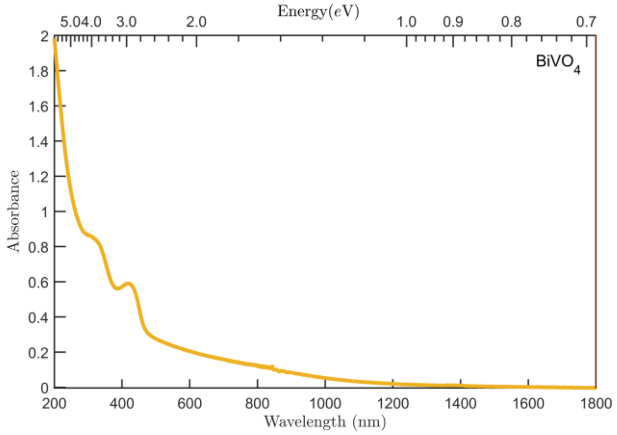

Fig. S5. Steady-state Uv-Vis-NIR absorption spectrum of $BiVO_4$ film.

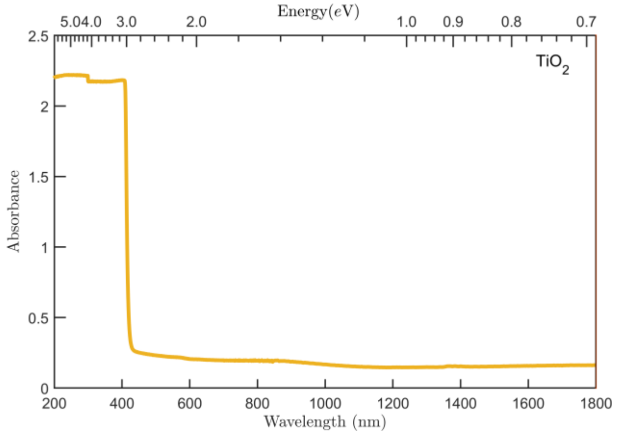

Fig. S6. Steady-state Uv-Vis-NIR absorption spectrum of TiO_2 single crystal.

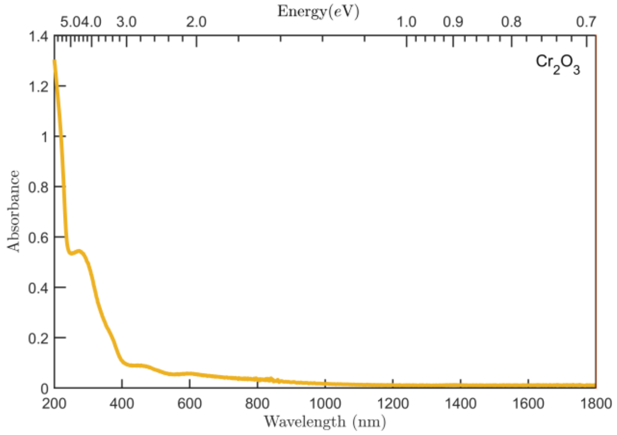

Fig. S7. Steady-state Uv-Vis-NIR absorption spectrum of Cr_2O_3 film.

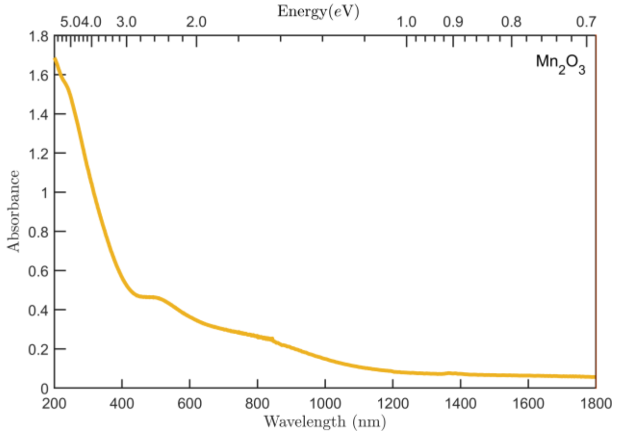

Fig. S8. Steady-state Uv-Vis-NIR absorption spectrum of Mn_2O_3 film.

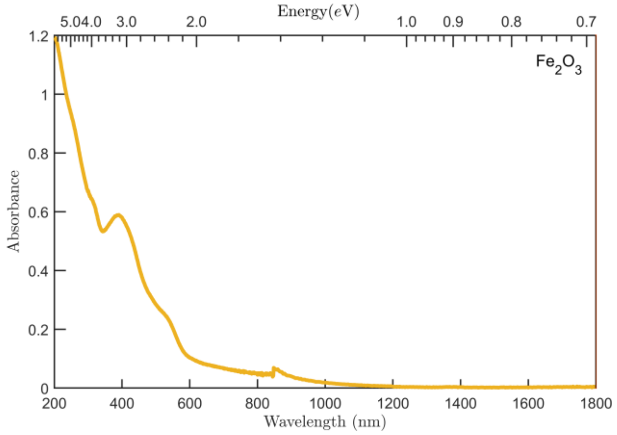

Fig. S9. Steady-state Uv-Vis-NIR absorption spectrum of Fe_2O_3 film.

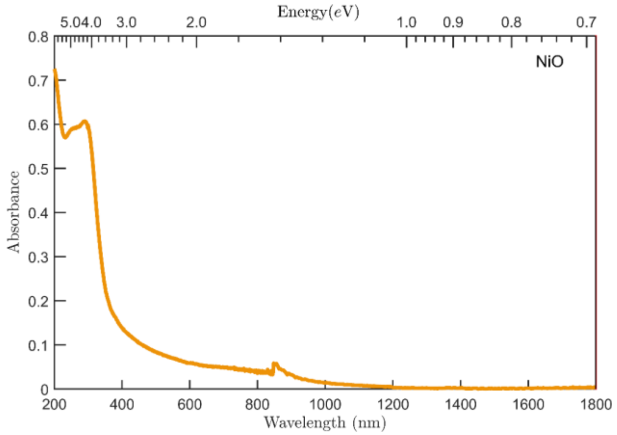

Fig. S10. Steady-state Uv-Vis-NIR absorption spectrum of NiO film.

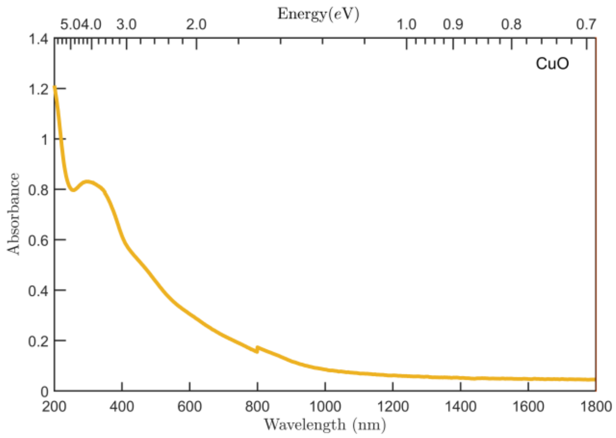

Fig. S11. Steady-state Uv-Vis-NIR absorption spectrum of CuO film.

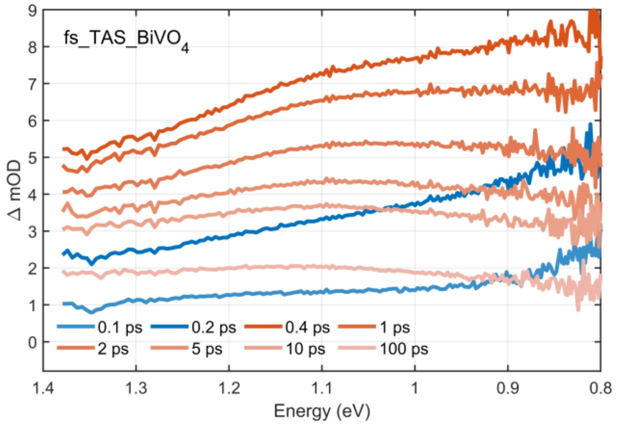

Fig. S12. The evolution of fs-TAS spectra collected on $BiVO_4$ film, with an excitation wavelength of 3.5 eV (355 nm, fluence of 2.5 mJ·cm^{-2}).

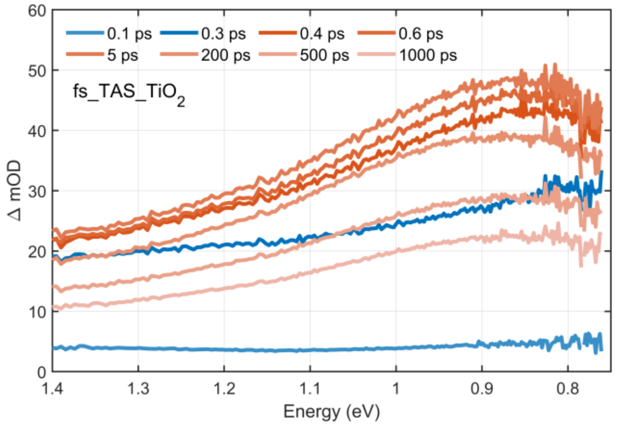

Fig. S13. The evolution of fs-TAS spectra collected on TiO_2 crystal, with an excitation wavelength of 3.5 eV (355 nm, fluence of 2.5 mJ·cm^{-2}).

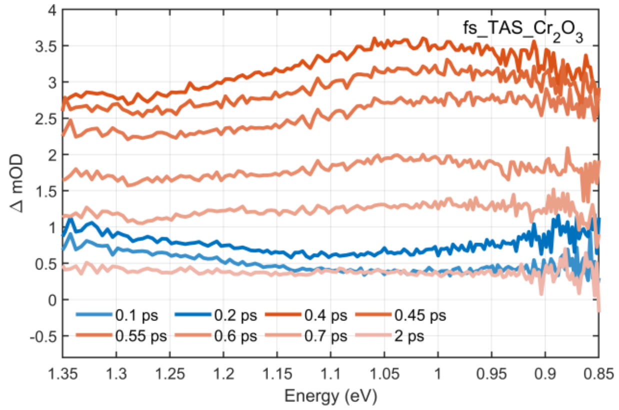

Fig. S14. The evolution of fs-TAS spectra collected on Cr_2O_3 film, with an excitation wavelength of 4.1 eV (305 nm, fluence of 2.2 mJ·cm^{-2}).

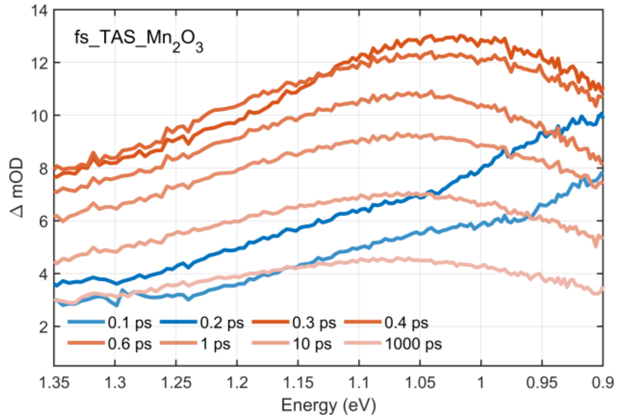

Fig. S15. The evolution of fs-TAS spectra collected on Mn_2O_3 film, with an excitation wavelength of 3.5 eV (355 nm, fluence of 2.3 mJ·cm^{-2}).

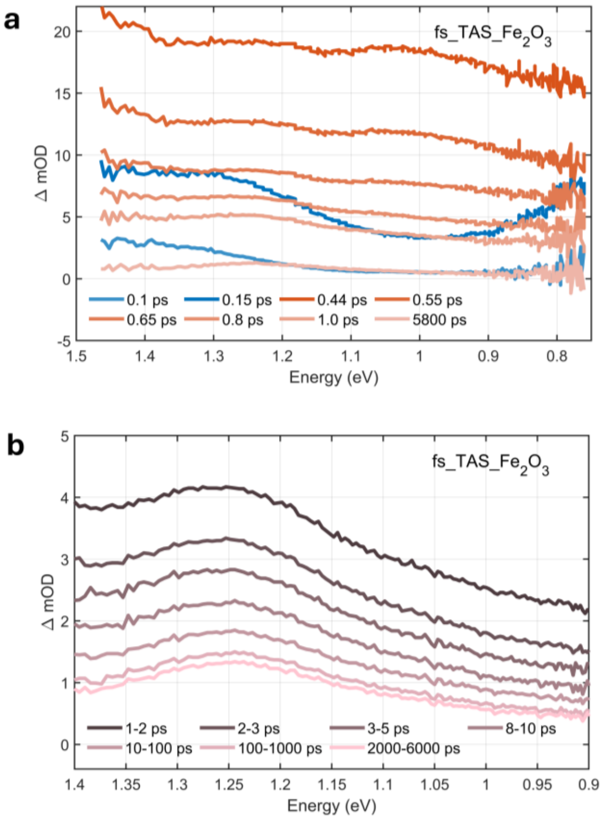

Fig. S16. The evolution of fs-TAS spectra collected on Fe_2O_3 film, with an excitation wavelength of 3.5 eV (355 nm, fluence of 2.5 mJ·cm^{-2}); **(a)** the full spectra; **(b)** the chosen spectra from 1 ps to 6000 ps. After 0.65 ps, only one peak was observed at 1.24 eV and kept decreasing until the end of the detection (see Fig. S16b).

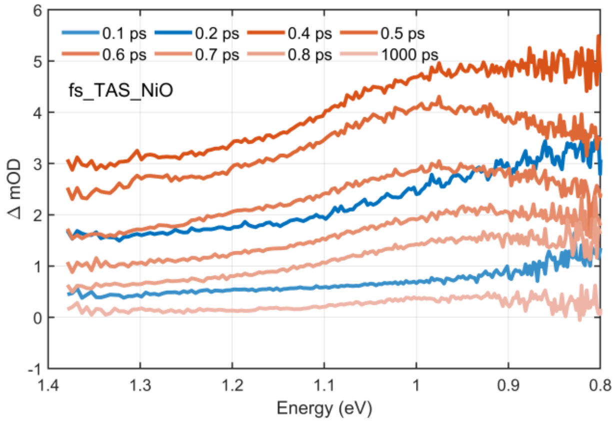

Fig. S17. The evolution of fs-TAS spectra collected on dense NiO film, with an excitation wavelength of 4.1 eV (305 nm, fluence of 2.1 mJ·cm^{-2}).

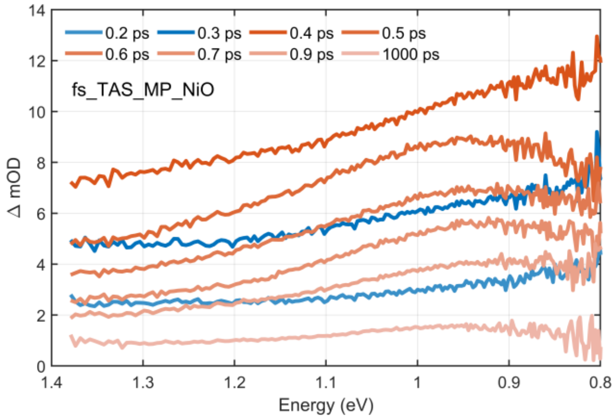

Fig. S18. The evolution of fs-TAS spectra collected on mesoporous NiO film, with an excitation wavelength of 3.5 eV (355 nm, fluence of 2.5 mJ·cm^{-2}). Similar fs-TAS evolution were observed in dense NiO and MP_NiO films, even in different film structures and excitation wavelengths.

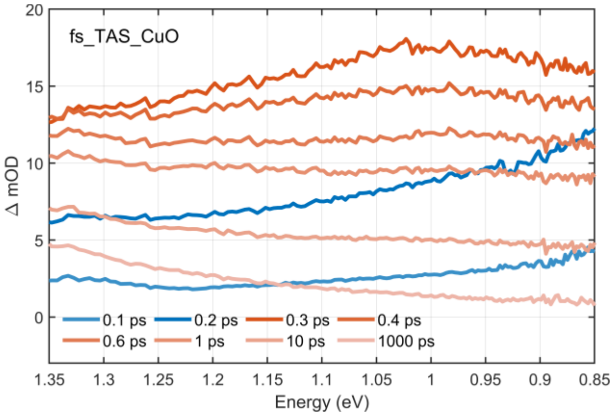

Fig. S19. The evolution of fs-TAS spectra collected on CuO film, with an excitation wavelength of 3.5 eV (355 nm, fluence of 2.9 mJ·cm^{-2}).

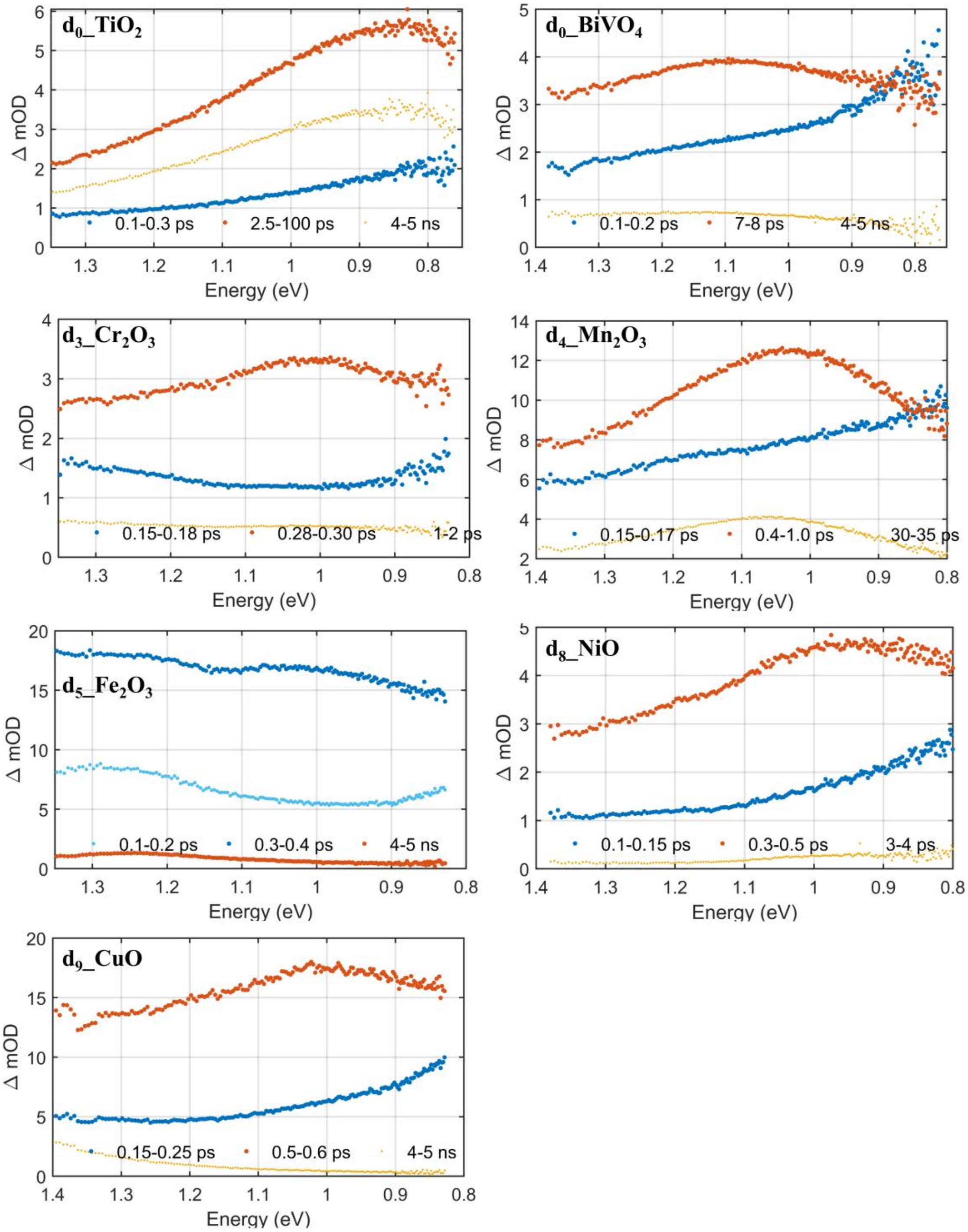

Fig. S20. Three representative averaged fs-TAS spectra chosen from the respective probe-delay windows in d_0-d_9 metal oxides studied.

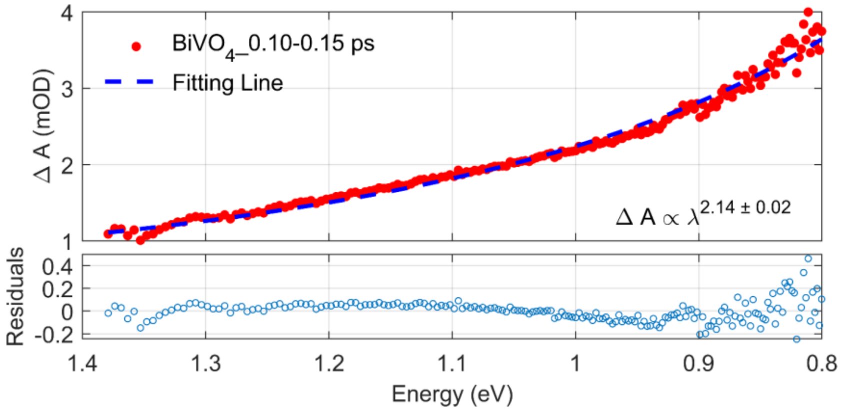

Fig. S21. Top: fs-TAS absorption of $BiVO_4$ film in the first 300 fs (red dots), with an excitation wavelength of 3.5 eV (355 nm, fluence of 2.5 mJ·cm^{-2}); Drude model fitting (blue dash line) and the fitted scaling exponents $\alpha = 2.14 \pm 0.02$ was inserted. Bottom: fitting residuals.

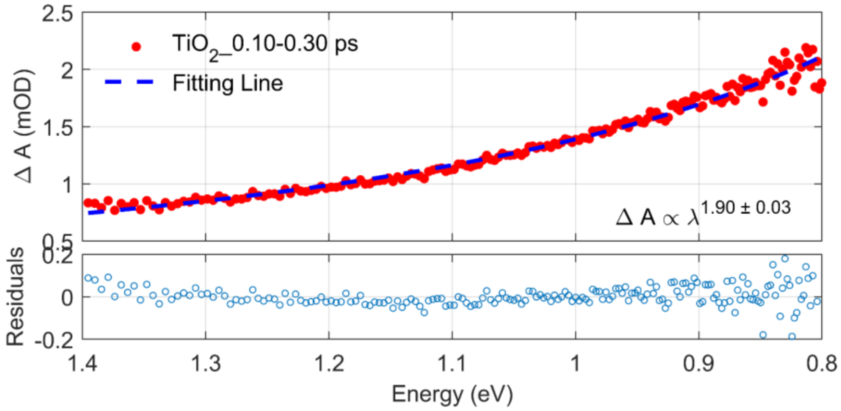

Fig. S22. Top: fs-TAS absorption of TiO_2 crystal in the first 300 fs (red dots), with an excitation wavelength of 3.5 eV (355 nm, fluence of 2.5 mJ·cm^{-2}); Drude model fitting (blue dash line) and the fitted scaling exponents $\alpha = 1.90 \pm 0.03$ was inserted. Bottom: fitting residuals.

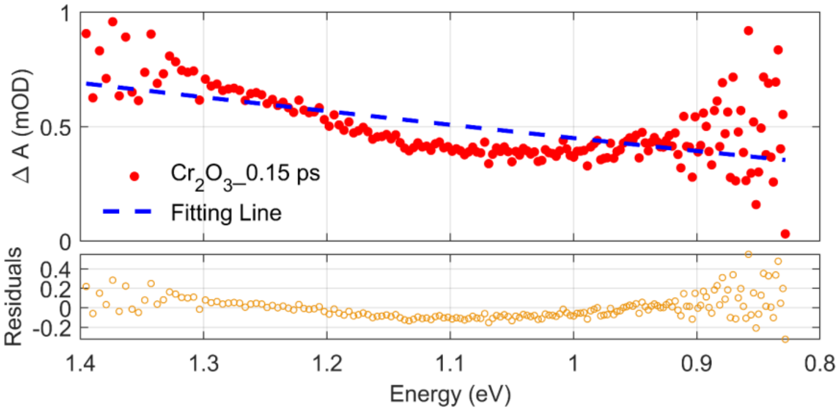

Fig. S23. Top: fs-TAS absorption of Cr_2O_3 film in the first 300 fs (red dots), with an excitation wavelength of 4.1 eV (305 nm, fluence of 2.2 mJ·cm^{-2}); In Cr_2O_3, Drude model fitting (blue dash line) is not successful, implying no free charge absorption observed in 100-300 fs. Bottom: fitting residuals.

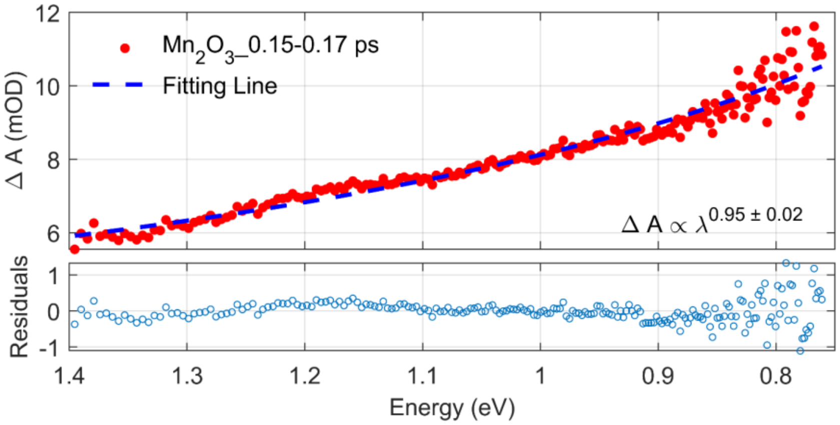

Fig. S24. Top: fs-TAS absorption of Mn_2O_3 film in the first 300 fs (red dots), with an excitation wavelength of 3.5 eV (355 nm, fluence of 2.3 mJ·cm^{-2}); Drude model fitting (blue dash line) and the fitted scaling exponents $\alpha = 0.95 \pm 0.02$ was inserted. Bottom: fitting residuals.

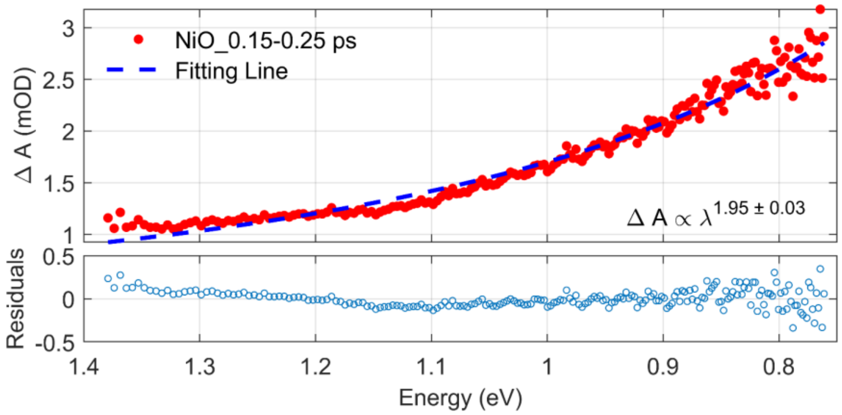

Fig. S25. Top: fs-TAS absorption of NiO dense film in the first 300 fs (red dots), with an excitation wavelength of 4.1 eV (305 nm, fluence of 2.1 mJ·cm^{-2}); Drude model fitting (blue dash line) and the fitted scaling exponents α = 1.95 ± 0.03 was inserted. Bottom: fitting residuals.

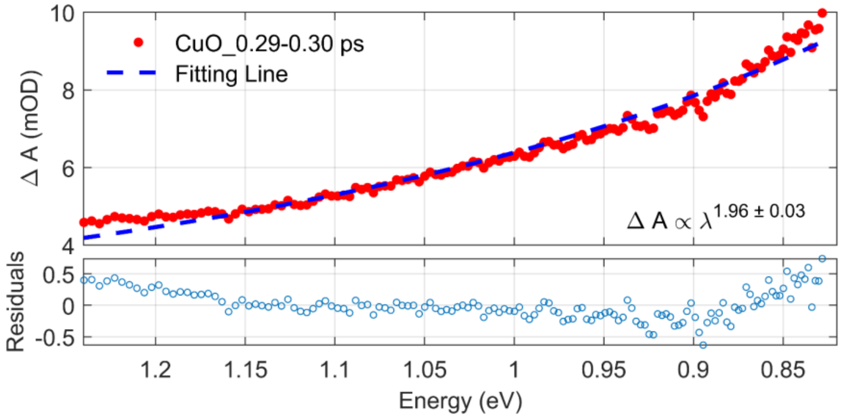

Fig. S26. Top: fs-TAS absorption of CuO film in the first 300 fs (red dots), with an excitation wavelength of 3.5 eV (355 nm, fluence of 2.9 mJ·cm^{-2}); Drude model fitting (blue dash line) and the fitted scaling exponents α = 1.96 ± 0.03 was inserted. Bottom: fitting residuals.

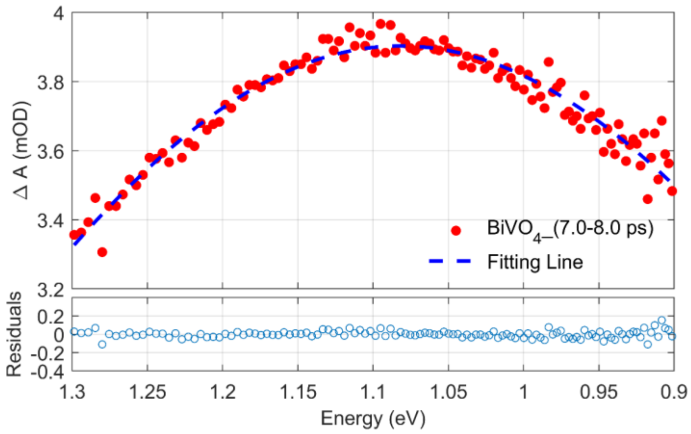

Fig. S27. Top: fs-TAS absorption of $BiVO_4$ film after free charge localization (red dots), with an excitation wavelength of 3.5 eV (355 nm, fluence of 2.5 mJ·cm^{-2}); The absorption spectrum was fitted with small polaron absorption model (blue dash line), and the fitted results were summarized in Table 1. Bottom: fitting residuals.

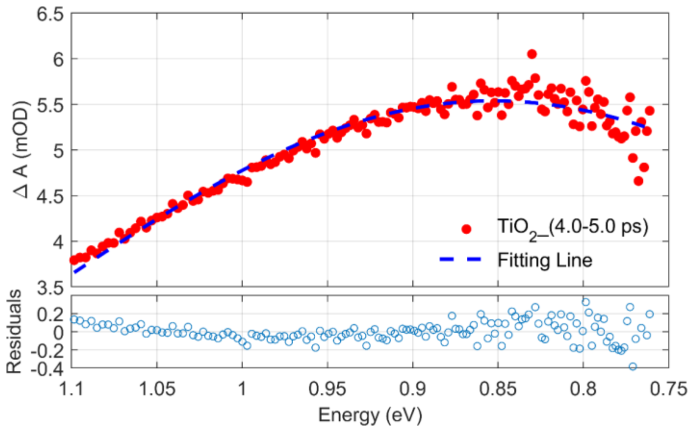

Fig. S28. Top: fs-TAS absorption of TiO_2 crystal after free charge localization (red dots), with an excitation wavelength of 3.5 eV (355 nm, fluence of 2.5 mJ·cm^{-2}); The absorption spectrum was fitted with small polaron absorption model (blue dash line), and the fitted results were summarized in Table 1. Bottom: fitting residuals.

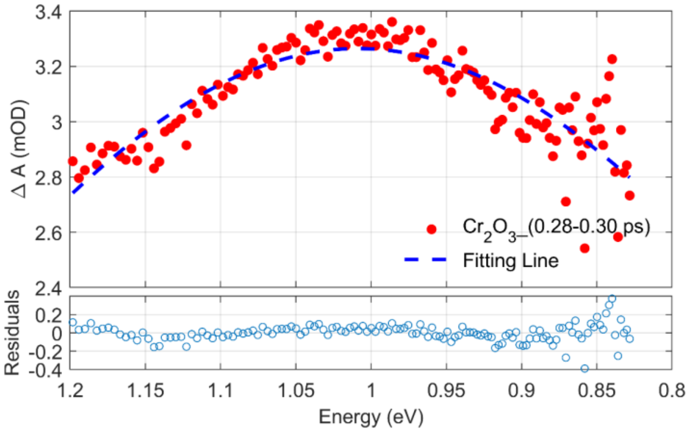

Fig. S29a. Top: fs-TAS absorption of Cr_2O_3 film after free charge localization (red dots), with an excitation wavelength of 3.8 eV (330 nm, fluence of 0.8 mJ·cm^{-2}); The absorption spectrum was fitted with small polaron absorption model (blue dash line). Bottom: fitting residuals.

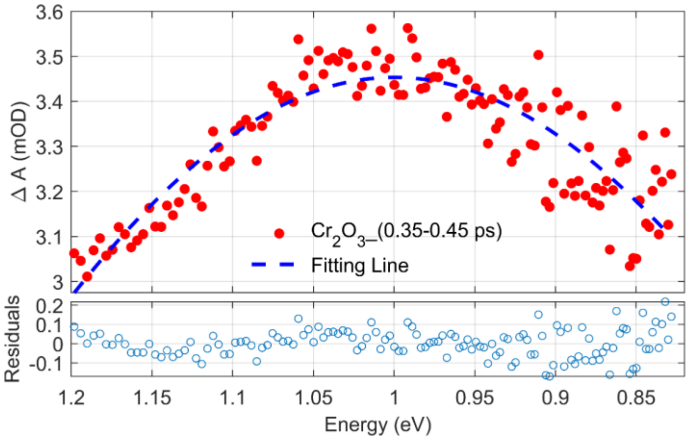

Fig. S29b. Top: fs-TAS absorption of Cr_2O_3 film after free charge localization (red dots), with an excitation wavelength of 4.1 eV (305 nm, fluence of 2.2 mJ·cm^{-2}); The absorption spectrum was fitted with small polaron absorption model (blue dash line), and the fitted results were summarized in Table 1. We use the result in Fig. S29a. to prove the peak observed in Fig. S29b. is reliable, even if the data are a bit scattered after 1 eV. Bottom: fitting residuals.

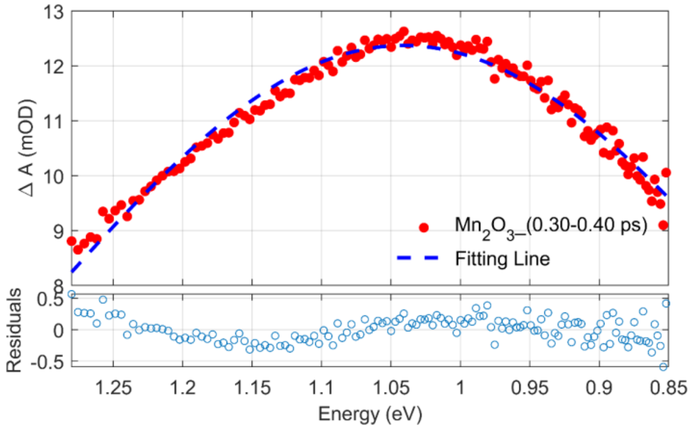

Fig. S30. Top: fs-TAS absorption of Mn_2O_3 film after free charge localization (red dots), with an excitation wavelength of 3.5 eV (355 nm, fluence of 2.3 mJ·cm^{-2}); The absorption spectrum was fitted with small polaron absorption model (blue dash line), and the fitted results were summarized in Table 1. Bottom: fitting residuals.

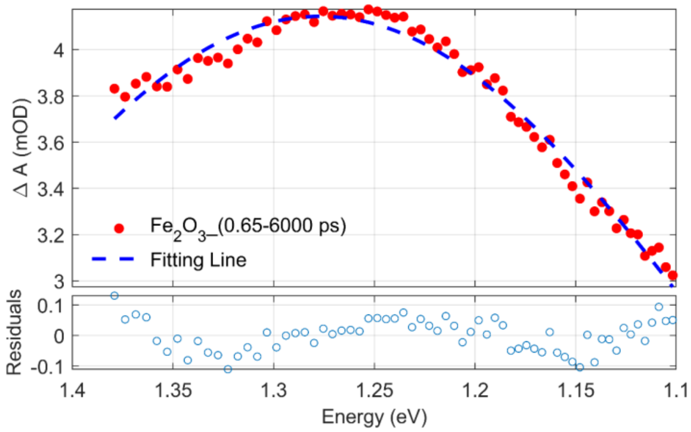

Fig. S31a. Top: fs-TAS absorption of Fe_2O_3 film after free charge localization (red dots), with an excitation wavelength of 3.5 eV (355 nm, fluence of 2.5 mJ·cm^{-2}); The absorption spectrum was fitted with small polaron absorption model (blue dash line), and the fitted results were summarized in Table 1. Bottom: fitting residuals.

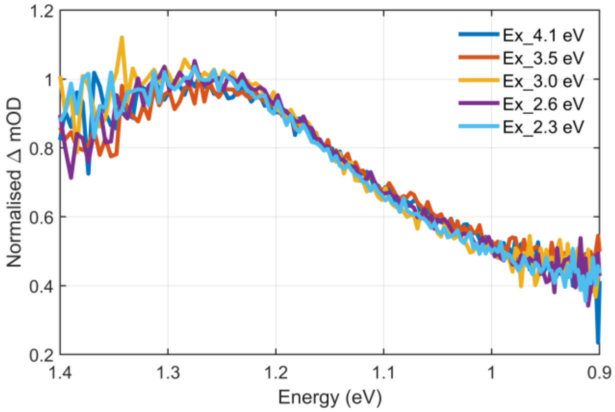

Fig. S31b. The excitation-energy dependence of the fs-TAS spectra of Fe_2O_3 acquired at 5ps.

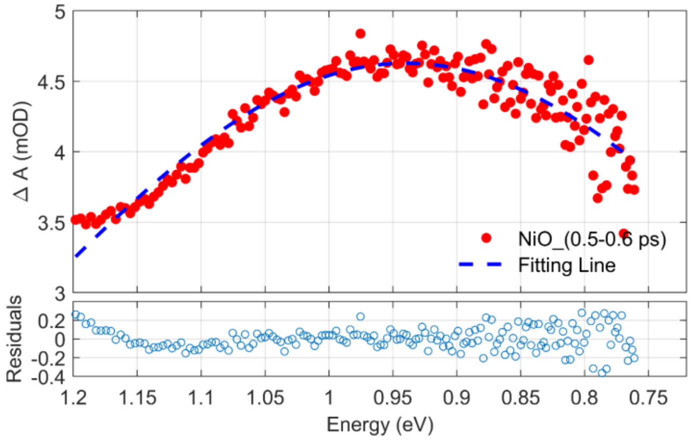

Fig. S32. Top: fs-TAS absorption of dense NiO film after free charge localization (red dots), with an excitation wavelength of 4.1 eV (305 nm, fluence of 2.1 mJ·cm^{-2}); The absorption spectrum was fitted with small polaron absorption model (blue dash line), and the fitted results were summarized in Table 1. Bottom: fitting residuals.

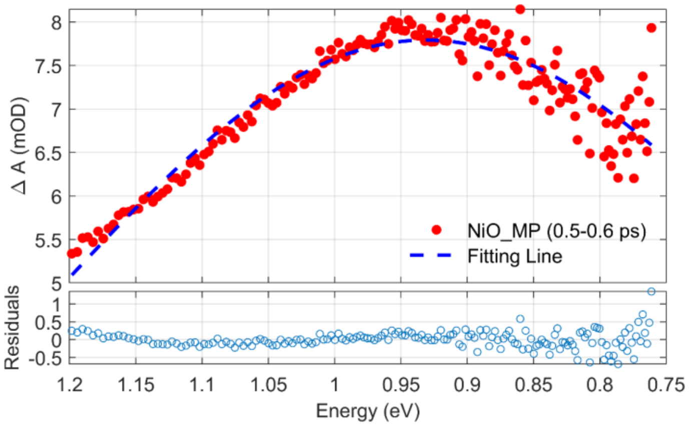

Fig. S33. Top: fs-TAS absorption of mesoporous NiO (NiO_MP) film after free charge localization (red dots), with an excitation wavelength of 4.1 eV (305 nm, fluence of 2.1 mJ·cm^{-2}); The absorption spectrum was fitted with small polaron absorption model (blue dash line), and the fitted results were summarized in Table S3. Bottom: fitting residuals. The mesoporous NiO_MP film was prepared based on previous publication[25].

Table S3. Comparison of small polaron absorptions from dense NiO thin film and mesoporous NiO film with rich defects.

		E_p (eV)	$\hbar w_0$ (eV)	E_a (eV)
d_8	NiO	0.52±0.001	0.084±0.002	0.26±0.001
d_8	NiO_MP	0.51±0.001	0.076±0.002	0.27±0.002

Even if Austin believed the absorption spectra observed in NiO should be attributed to trapped small hole polaron resulting from doped Li defects[26], we argued, in this scenario, the optical absorption spectra will shift while altering the defects origins. However, the abroad optical absorption still centred at 0.95 eV, even in a well-reported mesoporous NiO film with rich Ni vacancies[25] (see Fig. S31).

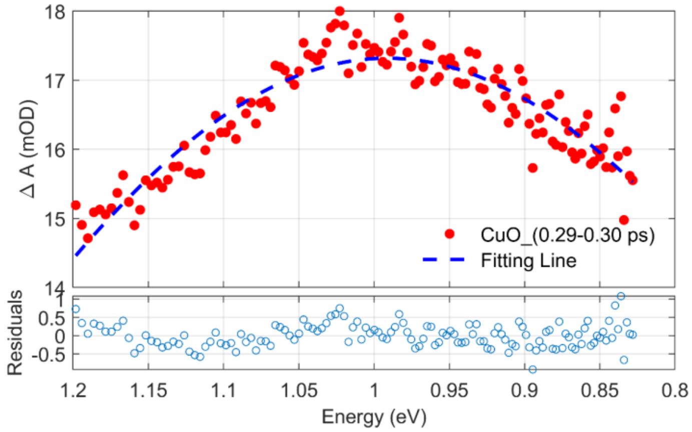

Fig. S34. Top: fs-TAS absorption of CuO film after free charge localization (red dots), with an excitation wavelength of 3.5 eV (355 nm, fluence of 2.9 mJ·cm^{-2}); The absorption spectrum was fitted with small polaron absorption model (blue dash line), and the fitted results were summarized in Table 1. Bottom: fitting residuals.

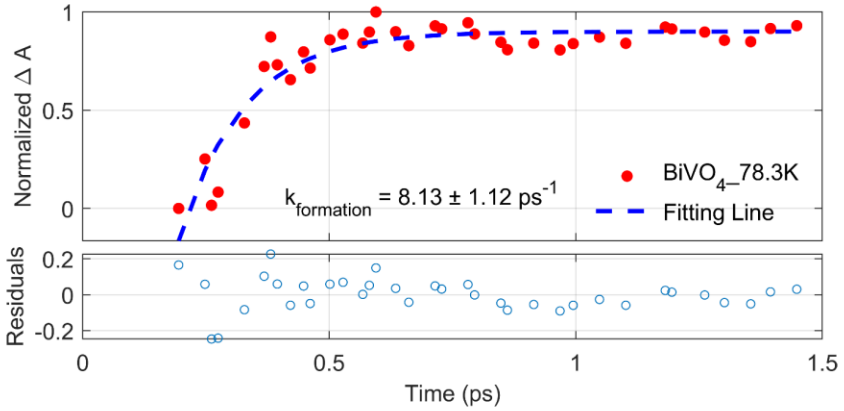

Fig. S35. Top: kinetic analysis of small polaron formation of $BiVO_4$ at 78.3 K with a single exponential fitting. The fitted rate constant was inserted in the plot. Bottom: fitting residuals.

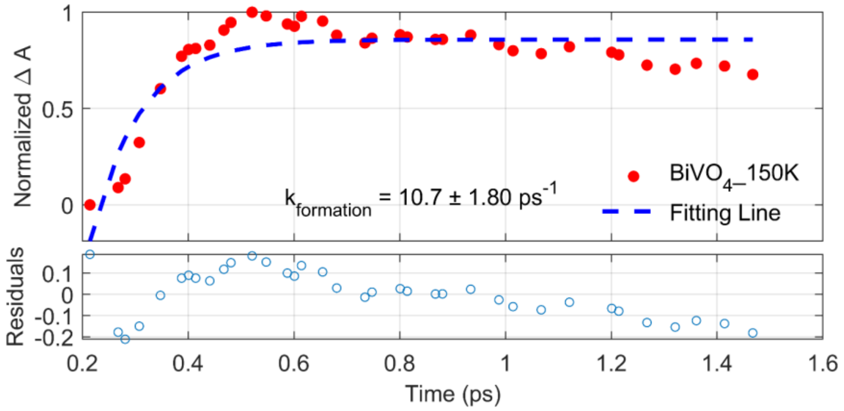

Fig. S36. Top: kinetic analysis of small polaron formation of $BiVO_4$ at 150 K with a single exponential fitting. The fitted rate constant was inserted in the plot. Bottom: fitting residuals.

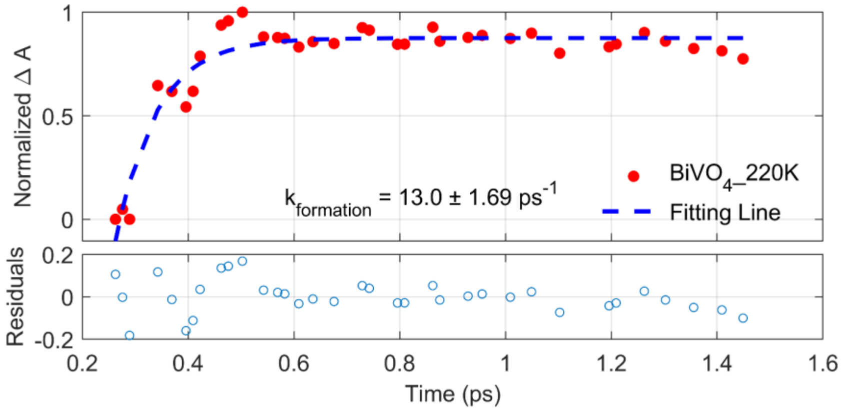

Fig. S37. Top: kinetic analysis of small polaron formation of $BiVO_4$ at 220 K with a single exponential fitting. The fitted rate constant was inserted in the plot. Bottom: fitting residuals.

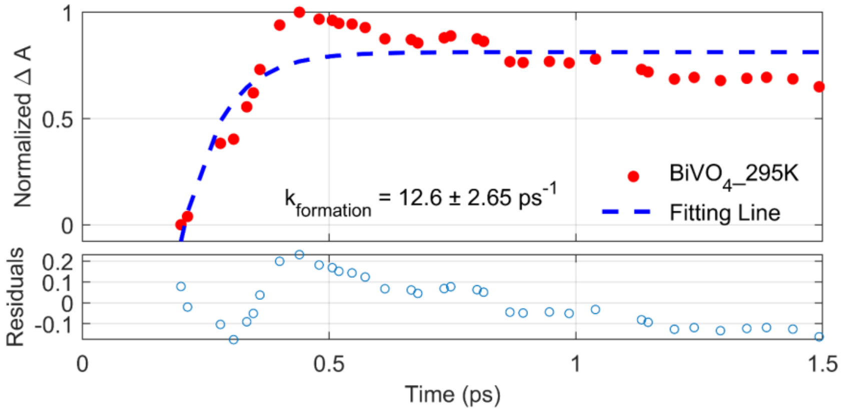

Fig. S38. Top: kinetic analysis of small polaron formation of $BiVO_4$ at 295 K with a single exponential fitting. The fitted rate constant was inserted in the plot. Bottom: fitting residuals.

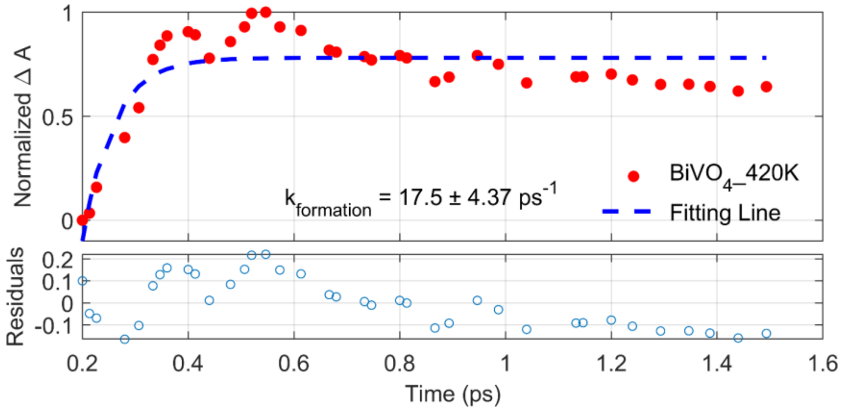

Fig. S39. Top: kinetic analysis of small polaron formation of $BiVO_4$ at 420 K with a single exponential fitting. The fitted rate constant was inserted in the plot. Bottom: fitting residuals.

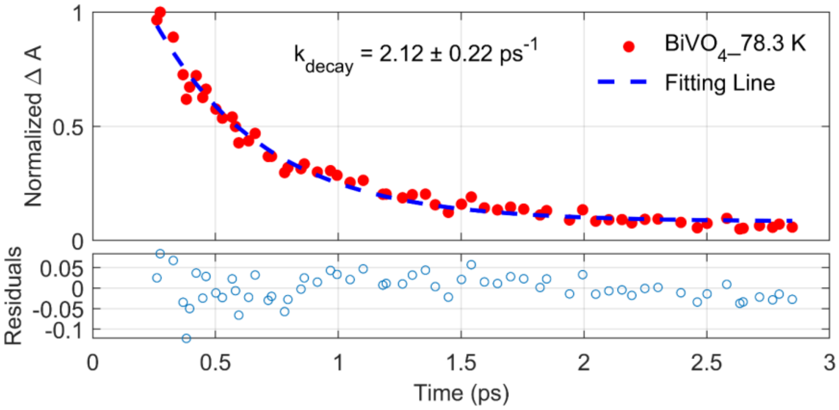

Fig. S40. Top: kinetic analysis of free electron decay of $BiVO_4$ at 78.3 K with a single exponential fitting. The fitted rate constant was inserted in the plot. Bottom: fitting residuals.

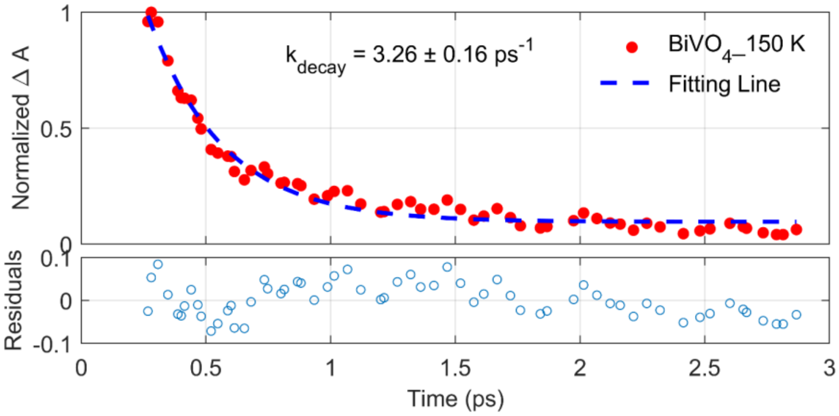

Fig. S41. Top: kinetic analysis of free electron decay of $BiVO_4$ at 150 K with a single exponential fitting. The fitted rate constant was inserted in the plot. Bottom: fitting residuals.

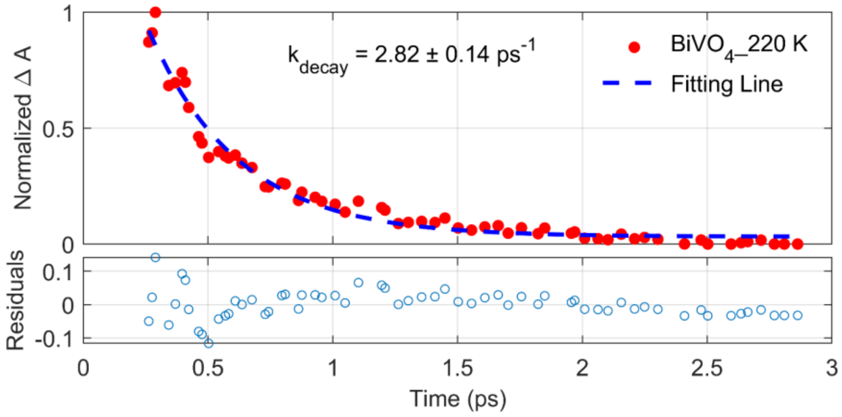

Fig. S42. Top: kinetic analysis of free electron decay of $BiVO_4$ at 220 K with a single exponential fitting. The fitted rate constant was inserted in the plot. Bottom: fitting residuals.

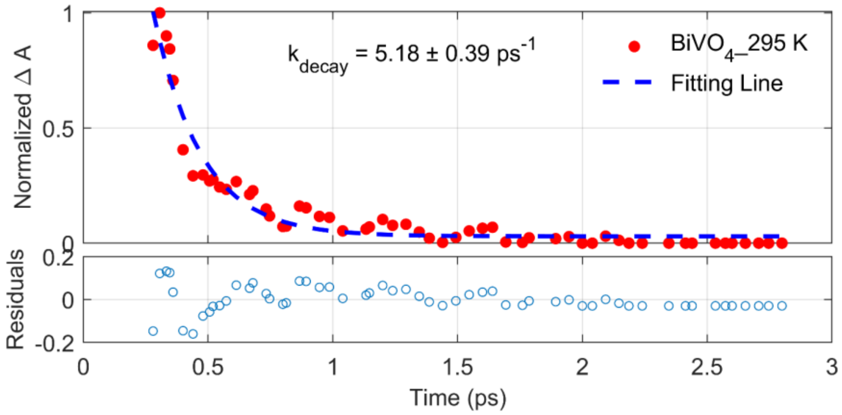

Fig. S43. Top: kinetic analysis of free electron decay of $BiVO_4$ at 295 K with a single exponential fitting. The fitted rate constant was inserted in the plot. Bottom: fitting residuals.

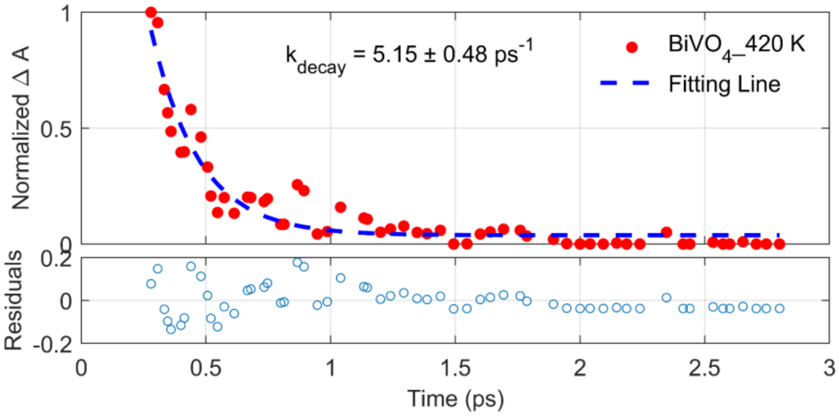

Fig. S44. Top: kinetic analysis of free electron decay of $BiVO_4$ at 420 K with a single exponential fitting. The fitted rate constant was inserted in the plot. Bottom: fitting residuals.

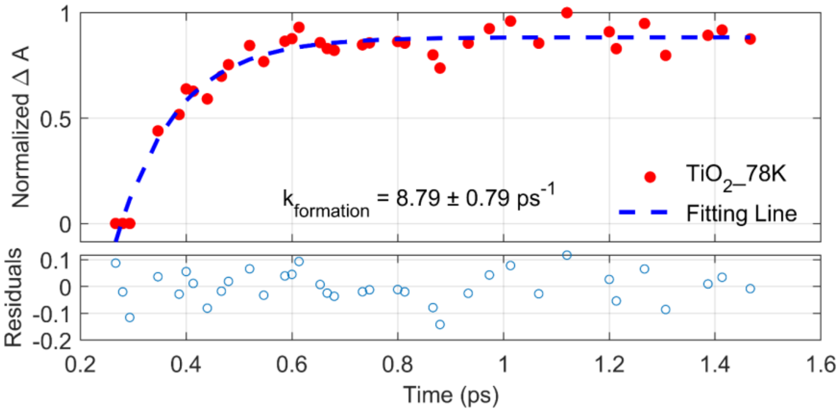

Fig. S45. Top: kinetic analysis of small polaron formation of TiO_2 at 78 K with a single exponential fitting. The fitted rate constant was inserted in the plot. Bottom: fitting residuals.

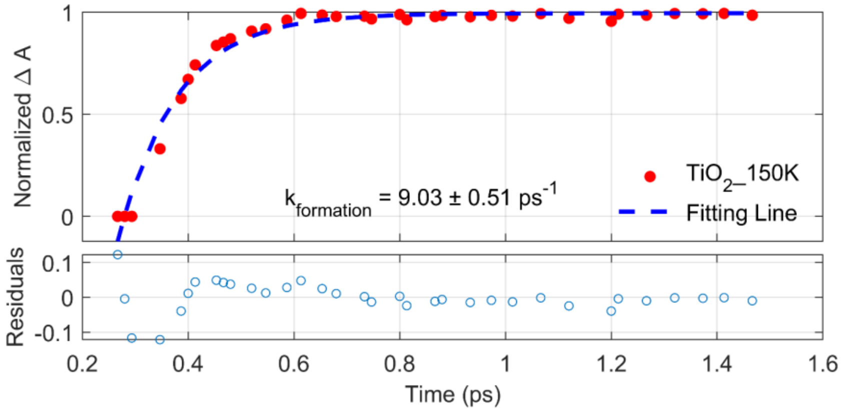

Fig. S46. Top: kinetic analysis of small polaron formation of TiO_2 at 150 K with a single exponential fitting. The fitted rate constant was inserted in the plot. Bottom: fitting residuals.

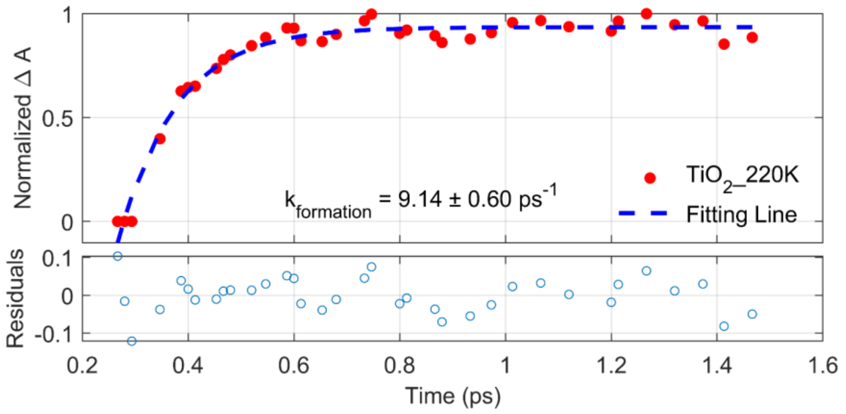

Fig. S47. Top: kinetic analysis of small polaron formation of TiO_2 at 220 K with a single exponential fitting. The fitted rate constant was inserted in the plot. Bottom: fitting residuals.

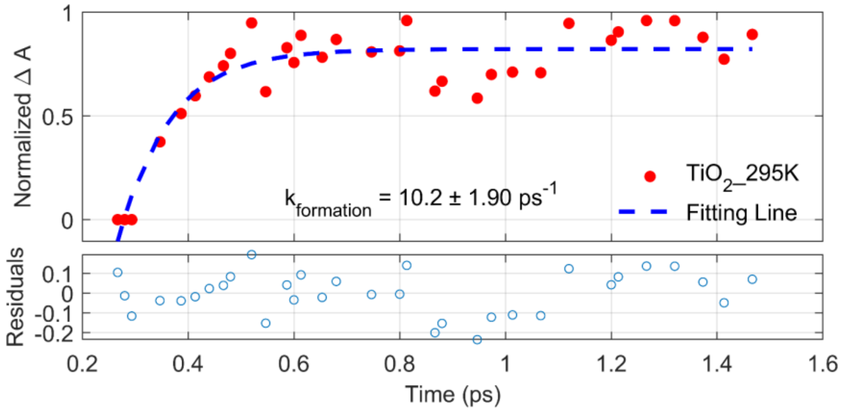

Fig. S48. Top: kinetic analysis of small polaron formation of TiO_2 at 295 K with a single exponential fitting. The fitted rate constant was inserted in the plot. Bottom: fitting residuals.

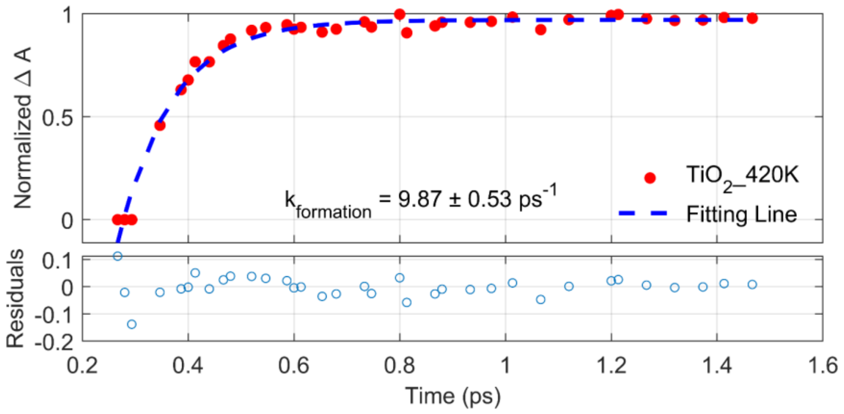

Fig. S49. Top: kinetic analysis of small polaron formation of TiO_2 at 420 K with a single exponential fitting. The fitted rate constant was inserted in the plot. Bottom: fitting residuals.

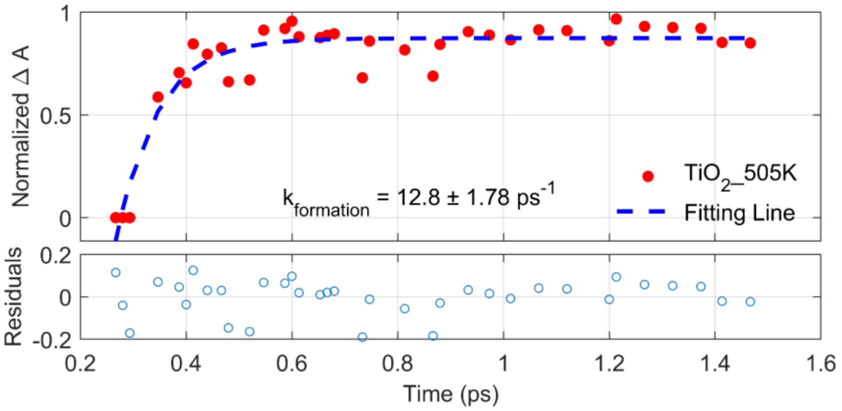

Fig. S50. Top: kinetic analysis of small polaron formation of TiO_2 at 505 K with a single exponential fitting. The fitted rate constant was inserted in the plot. Bottom: fitting residuals.

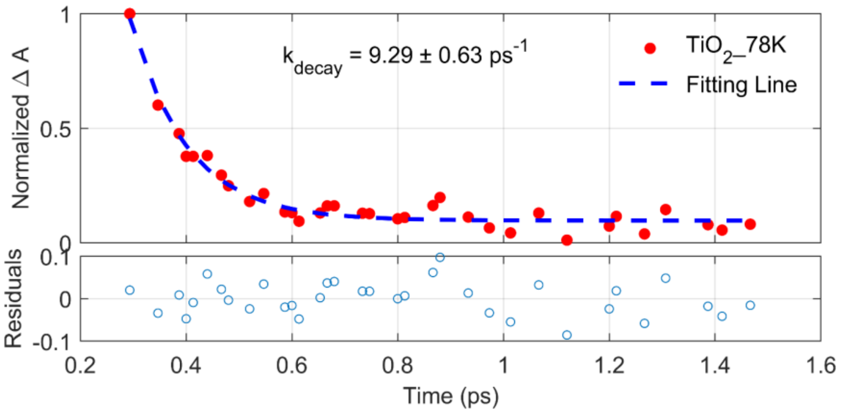

Fig. S51. Top: kinetic analysis of free charge decay of TiO_2 at 78 K with a single exponential fitting. The fitted rate constant was inserted in the plot. Bottom: fitting residuals.

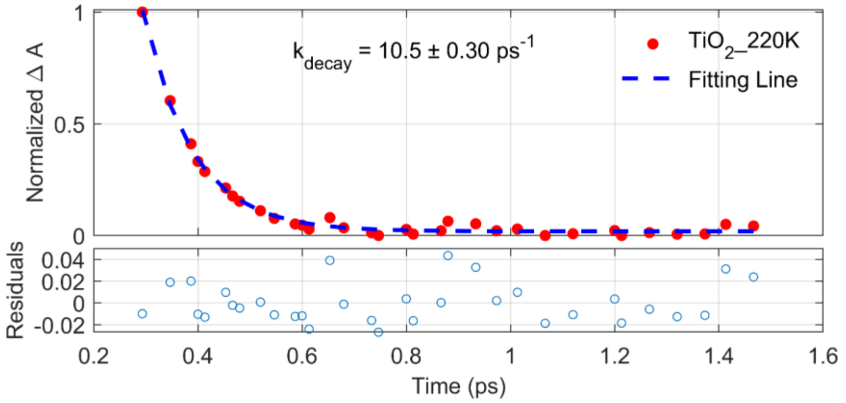

Fig. S52. Top: kinetic analysis of free charge decay of TiO_2 at 220 K with a single exponential fitting. The fitted rate constant was inserted in the plot. Bottom: fitting residuals.

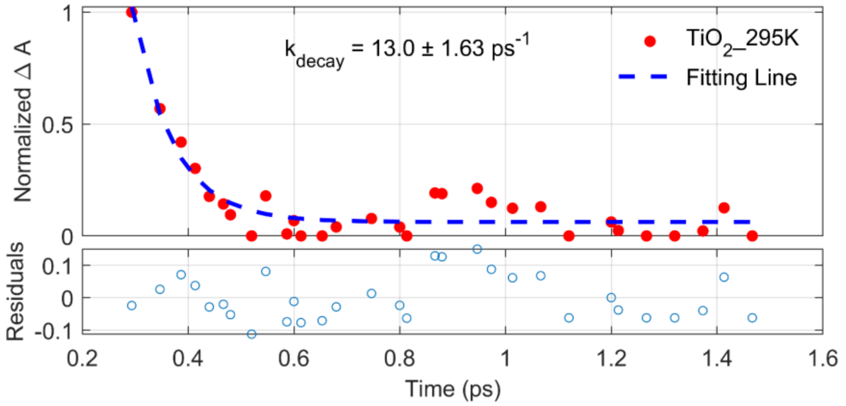

Fig. S53. Top: kinetic analysis of free charge decay of TiO_2 at 295 K with a single exponential fitting. The fitted rate constant was inserted in the plot. Bottom: fitting residuals.

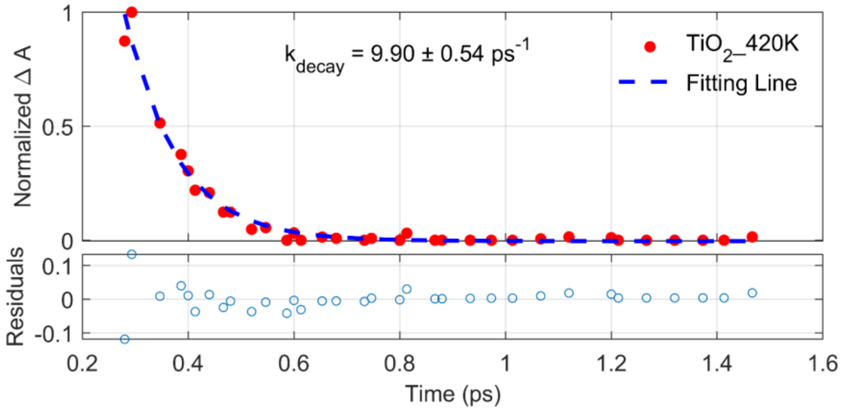

Fig. S54. Top: kinetic analysis of free charge decay of TiO_2 at 420 K with a single exponential fitting. The fitted rate constant was inserted in the plot. Bottom: fitting residuals.

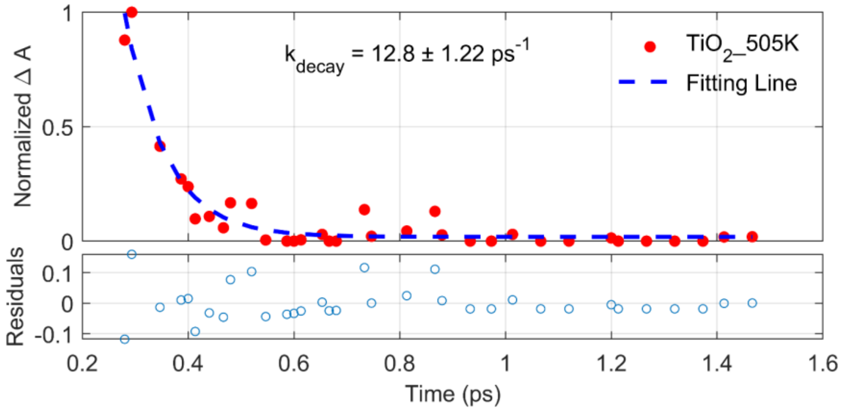

Fig. S55. Top: kinetic analysis of free charge decay of TiO_2 at 505 K with a single exponential fitting. The fitted rate constant was inserted in the plot. Bottom: fitting residuals.

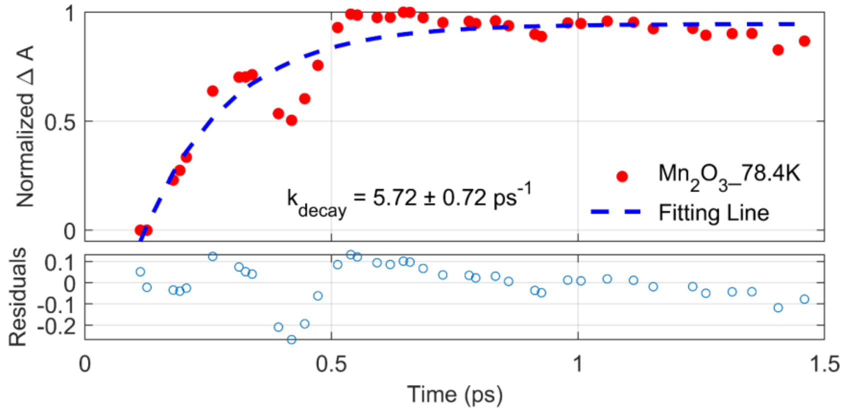

Fig. S56. Top: kinetic analysis of small polaron formation of Mn_2O_3 at 78.4 K with a single exponential fitting. The fitted rate constant was inserted in the plot. Bottom: fitting residuals.

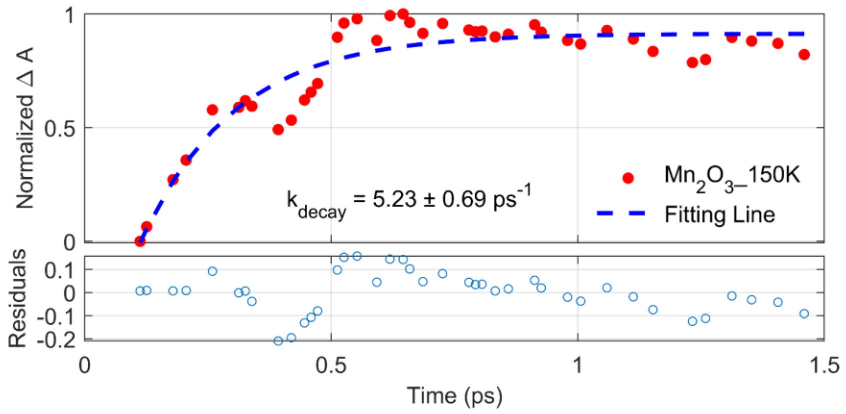

Fig. S57. Top: kinetic analysis of small polaron formation of Mn_2O_3 at 150 K with a single exponential fitting. The fitted rate constant was inserted in the plot. Bottom: fitting residuals.

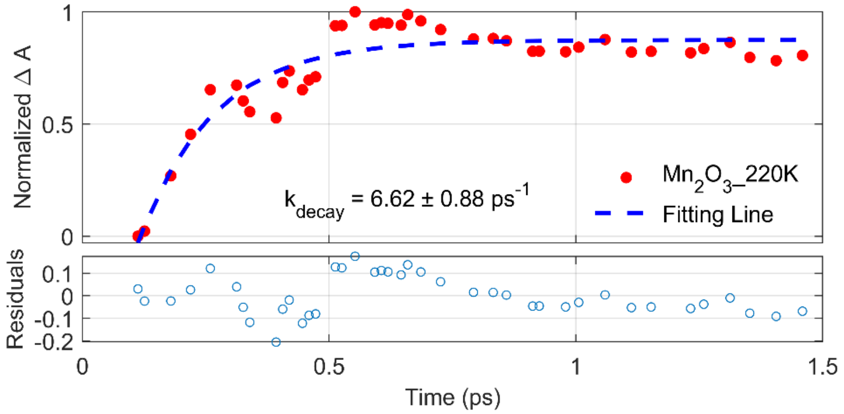

Fig. S58. Top: kinetic analysis of small polaron formation of Mn_2O_3 at 220 K with a single exponential fitting. The fitted rate constant was inserted in the plot. Bottom: fitting residuals.

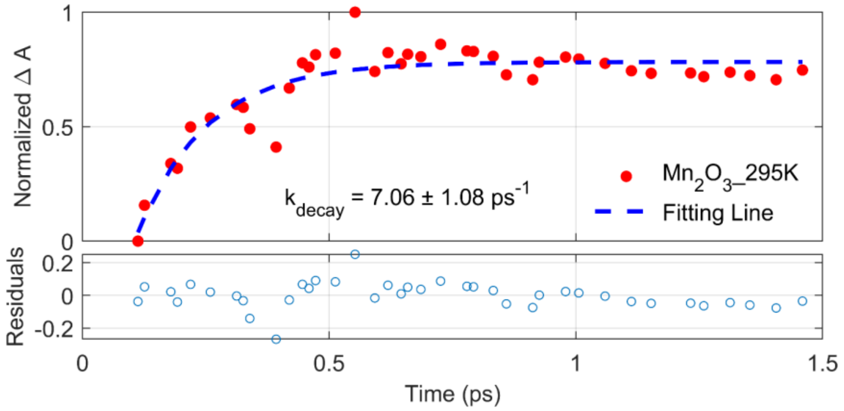

Fig. S59. Top: kinetic analysis of small polaron formation of Mn_2O_3 at 295 K with a single exponential fitting. The fitted rate constant was inserted in the plot. Bottom: fitting residuals.

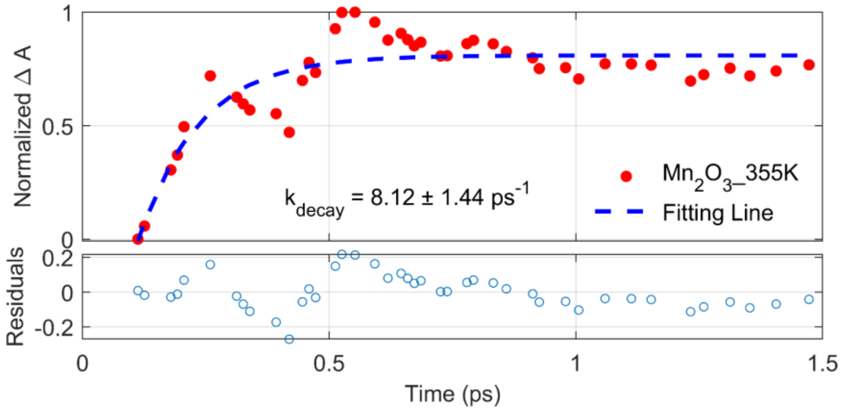

Fig. S60. Top: kinetic analysis of small polaron formation of Mn_2O_3 at 355 K with a single exponential fitting. The fitted rate constant is inserted in the plot. Bottom: fitting residuals.

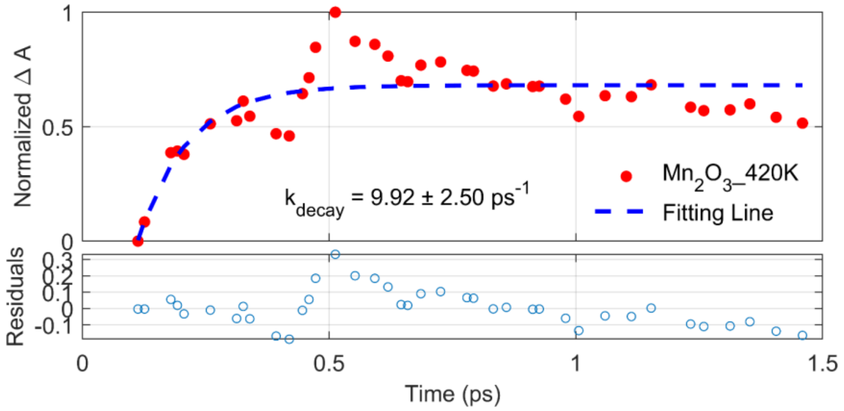

Fig. S61. Top: kinetic analysis of small polaron formation of Mn_2O_3 at 420 K with a single exponential fitting. The fitted rate constant was inserted in the plot. Bottom: fitting residuals.

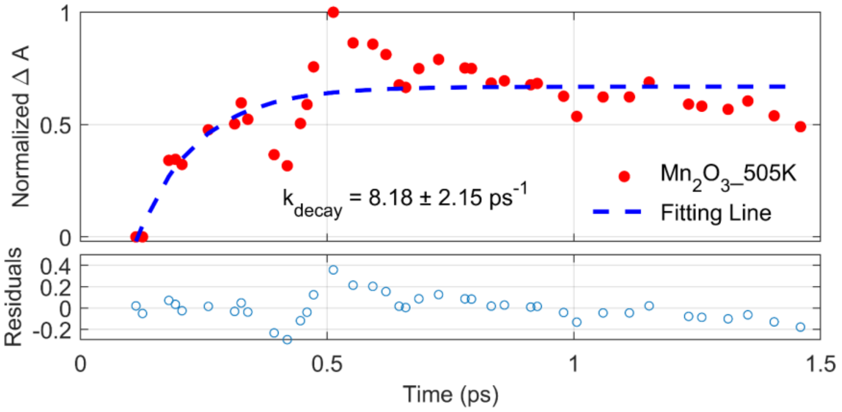

Fig. S62. Top: kinetic analysis of small polaron formation of Mn_2O_3 at 505 K with a single exponential fitting. The fitted rate constant was inserted in the plot. Bottom: fitting residuals.

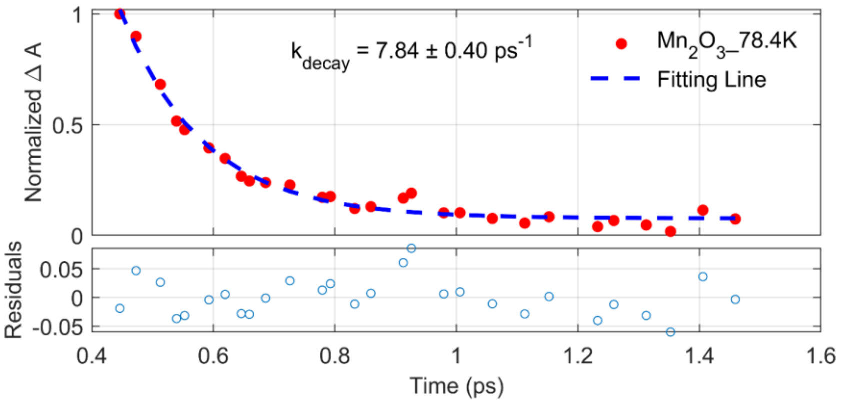

Fig. S63. Top: kinetic analysis of free charge decay of Mn_2O_3 at 78.4 K with a single exponential fitting. The fitted rate constant was inserted in the plot. Bottom: fitting residuals.

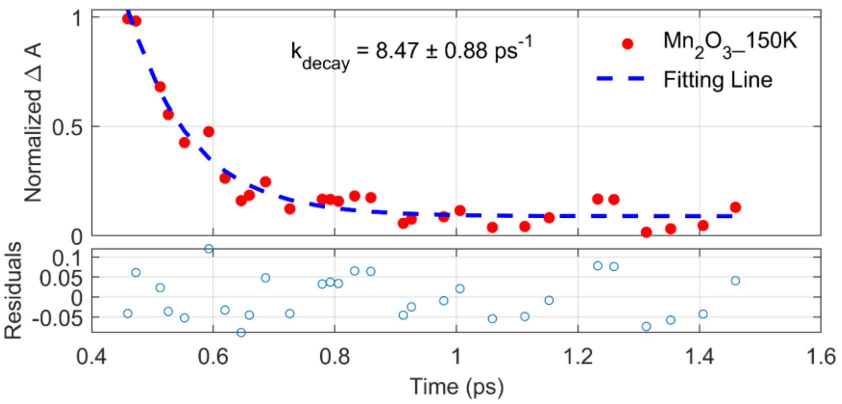

Fig. S64. Top: kinetic analysis of free charge decay of Mn_2O_3 at 150 K with a single exponential fitting. The fitted rate constant was inserted in the plot. Bottom: fitting residuals.

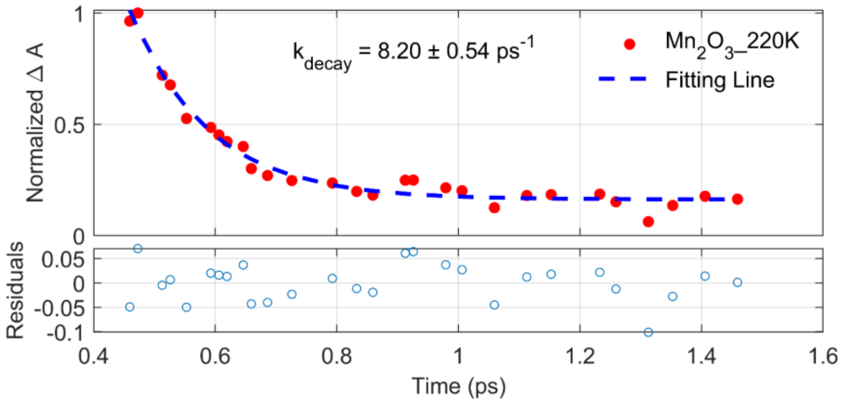

Fig. S65. Top: kinetic analysis of free charge decay of Mn_2O_3 at 220 K with a single exponential fitting. The fitted rate constant was inserted in the plot. Bottom: fitting residuals.

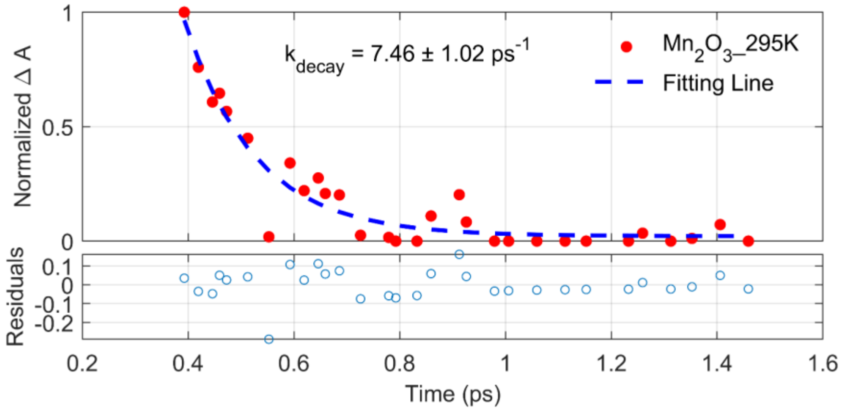

Fig. S66. Top: kinetic analysis of free charge decay of Mn_2O_3 at 295 K with a single exponential fitting. The fitted rate constant was inserted in the plot. Bottom: fitting residuals.

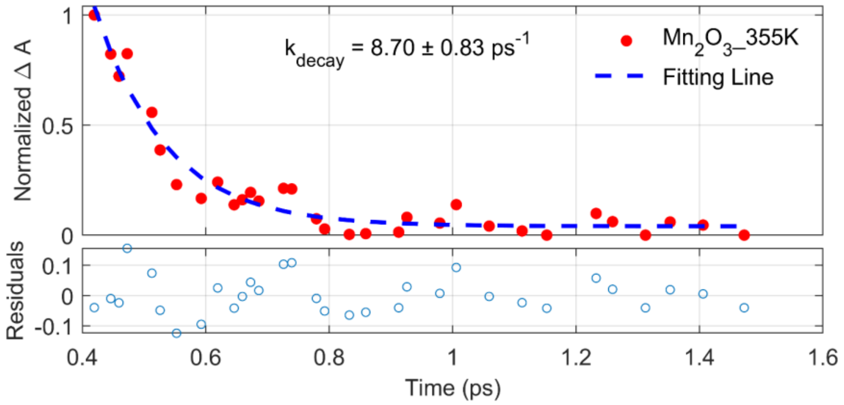

Fig. S67. Top: kinetic analysis of free charge decay of Mn_2O_3 at 355 K with a single exponential fitting. The fitted rate constant was inserted in the plot. Bottom: fitting residuals.

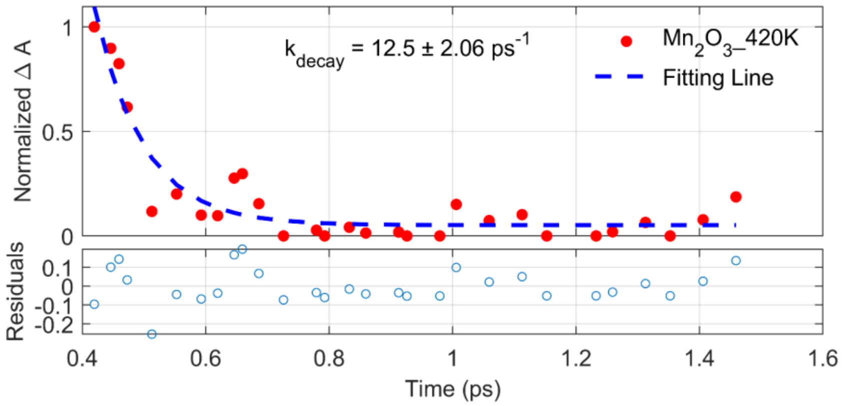

Fig. S68. Top: kinetic analysis of free charge decay of Mn_2O_3 at 7420 K with a single exponential fitting. The fitted rate constant was inserted in the plot. Bottom: fitting residuals.

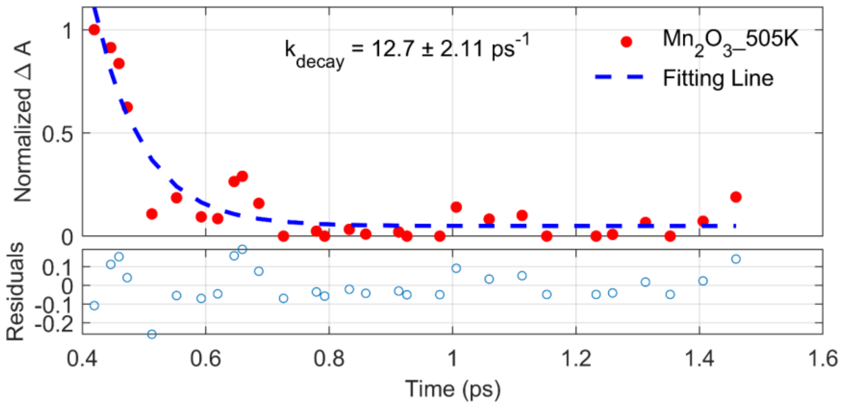

Fig. S69. Top: kinetic analysis of free charge decay of Mn_2O_3 at 505 K with a single exponential fitting. The fitted rate constant was inserted in the plot. Bottom: fitting residuals.

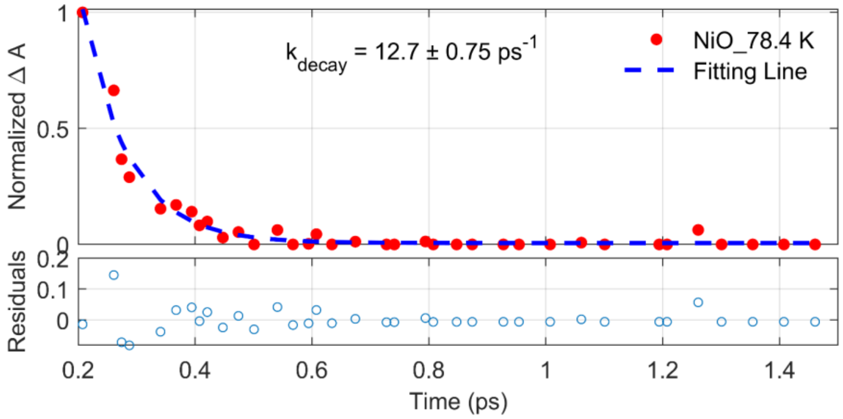

Fig. S70. Top: kinetic analysis of free charge decay of NiO at 78.4 K with a single exponential fitting. The fitted rate constant was inserted in the plot. Bottom: fitting residuals.

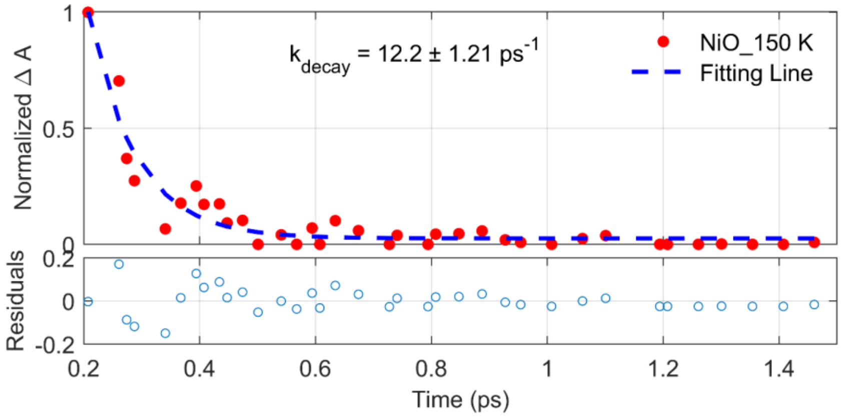

Fig. S71. Top: kinetic analysis of free charge decay of NiO at 150 K with a single exponential fitting. The fitted rate constant was inserted in the plot. Bottom: fitting residuals.

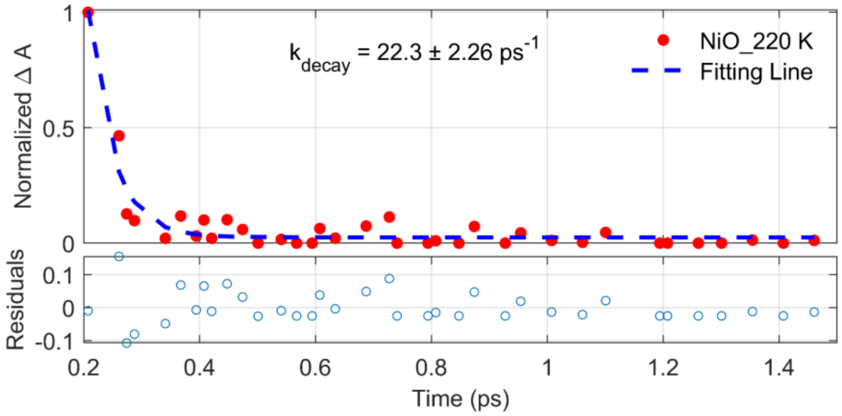

Fig. S72. Top: kinetic analysis of free charge decay of NiO at 220 K with a single exponential fitting. The fitted rate constant was inserted in the plot. Bottom: fitting residuals.

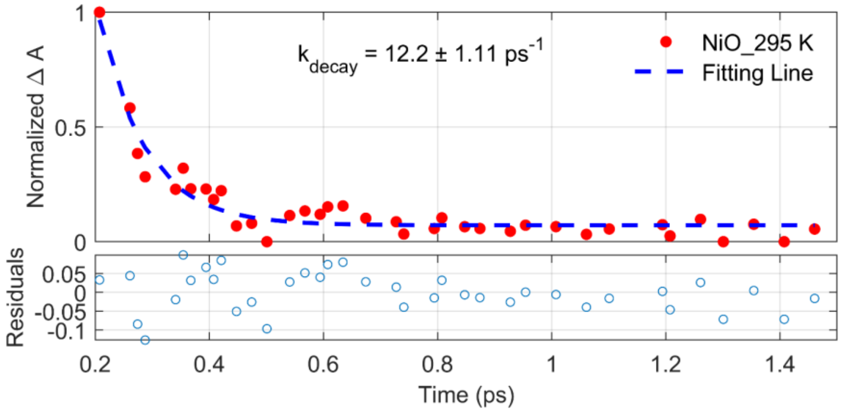

Fig. S73. Top: kinetic analysis of free charge decay of NiO at 295 K with a single exponential fitting. The fitted rate constant was inserted in the plot. Bottom: fitting residuals.

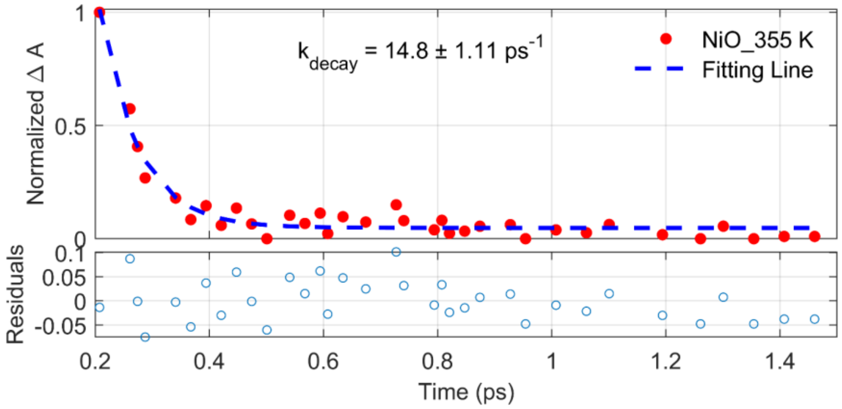

Fig. S74. Top: kinetic analysis of free charge decay of NiO at 355 K with a single exponential fitting. The fitted rate constant was inserted in the plot. Bottom: fitting residuals.

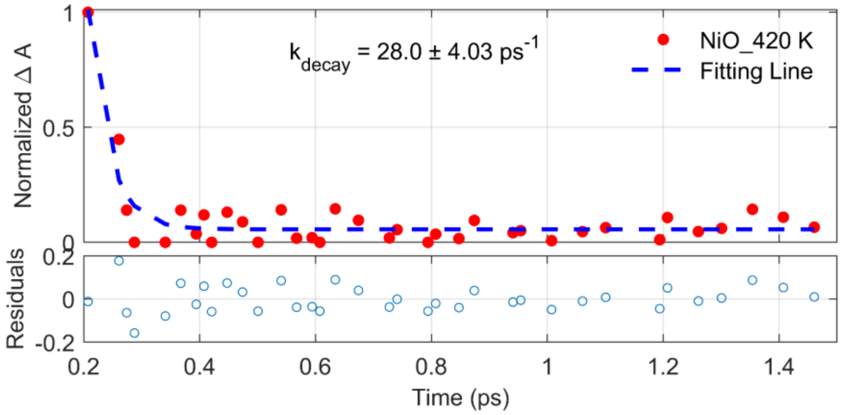

Fig. S75. Top: kinetic analysis of free charge decay of NiO at 420 K with a single exponential fitting. The fitted rate constant was inserted in the plot. Bottom: fitting residuals.

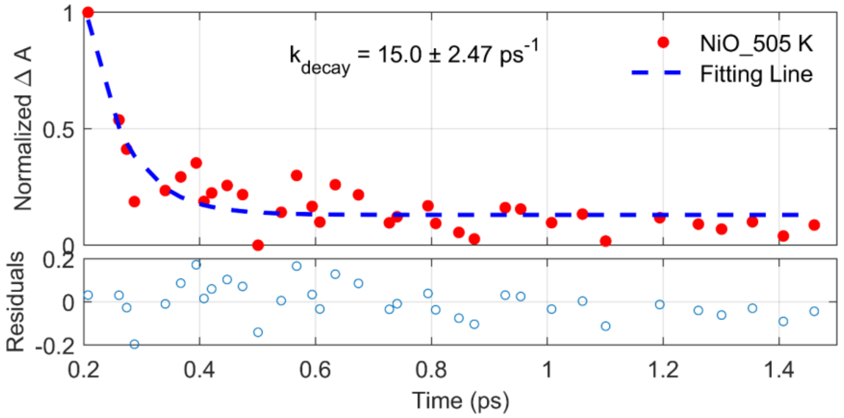

Fig. S76. Top: kinetic analysis of free charge decay of NiO at 505 K with a single exponential fitting. The fitted rate constant was inserted in the plot. Bottom: fitting residuals.

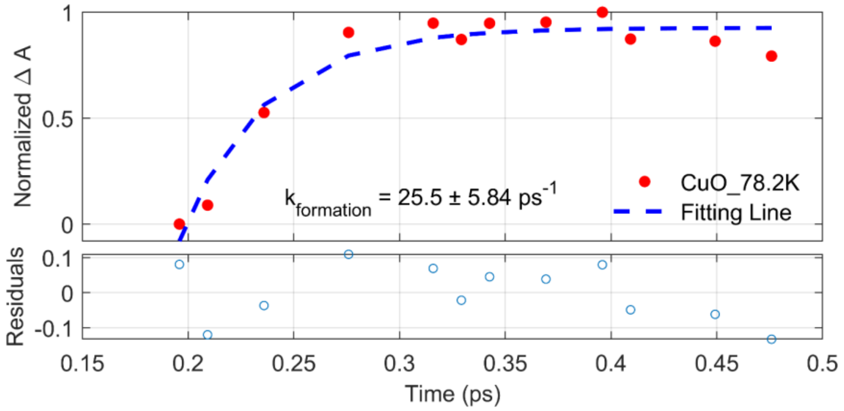

Fig. S77. Top: kinetic analysis of small polaron formation at 78.2 K with a single exponential fitting. The fitted rate constant was inserted in the plot. Bottom: fitting residuals.

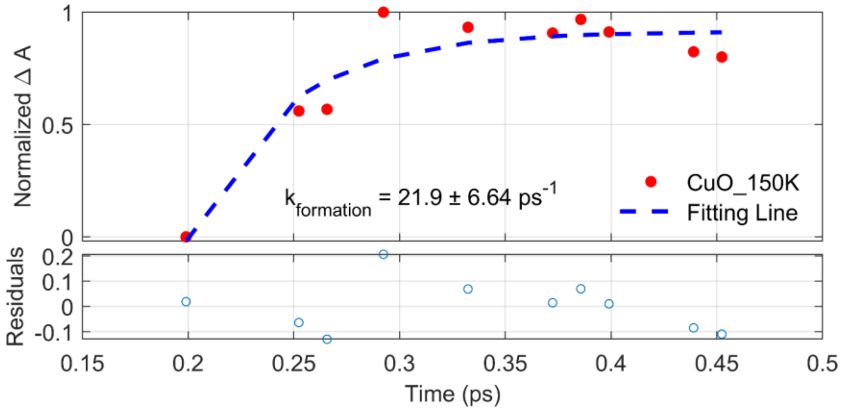

Fig. S78. Top: kinetic analysis of small polaron formation at 150 K with a single exponential fitting. The fitted rate constant was inserted in the plot. Bottom: fitting residuals.

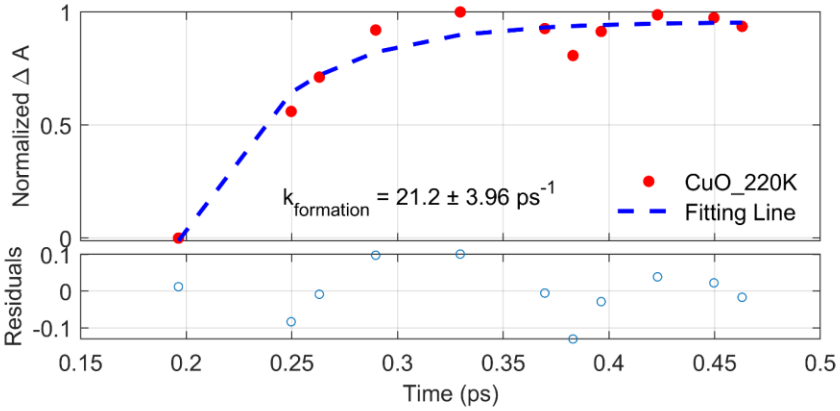

Fig. S79. Top: kinetic analysis of small polaron formation at 220 K with a single exponential fitting. The fitted rate constant was inserted in the plot. Bottom: fitting residuals.

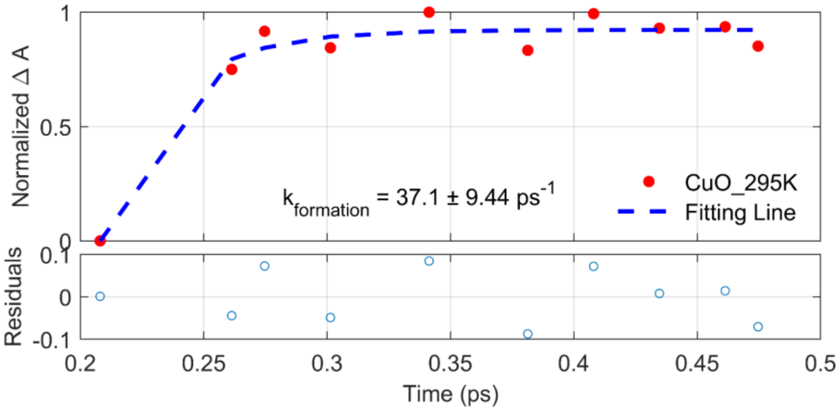

Fig. S80. Top: kinetic analysis of small polaron formation at 295 K with a single exponential fitting. The fitted rate constant was inserted in the plot. Bottom: fitting residuals.

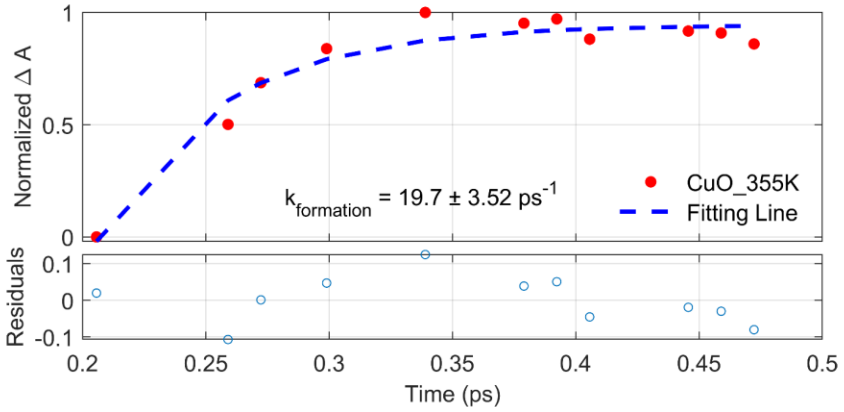

Fig. S81. Top: kinetic analysis of small polaron formation at 355 K with a single exponential fitting. The fitted rate constant was inserted in the plot. Bottom: fitting residuals.

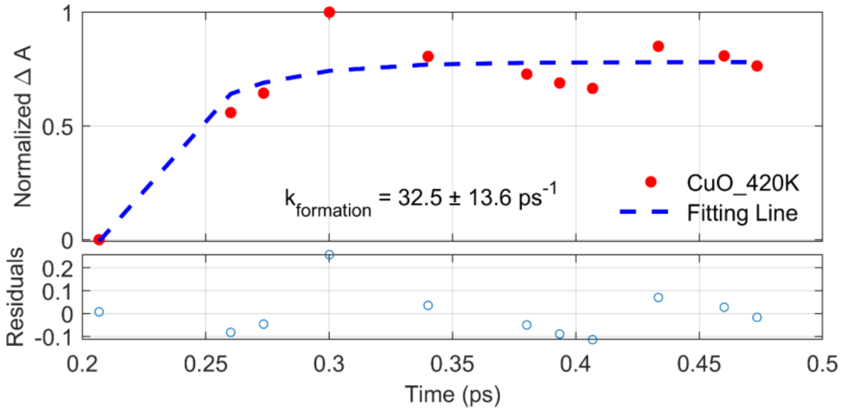

Fig. S82. Top: kinetic analysis of small polaron formation at 420 K with a single exponential fitting. The fitted rate constant was inserted in the plot. Bottom: fitting residuals.

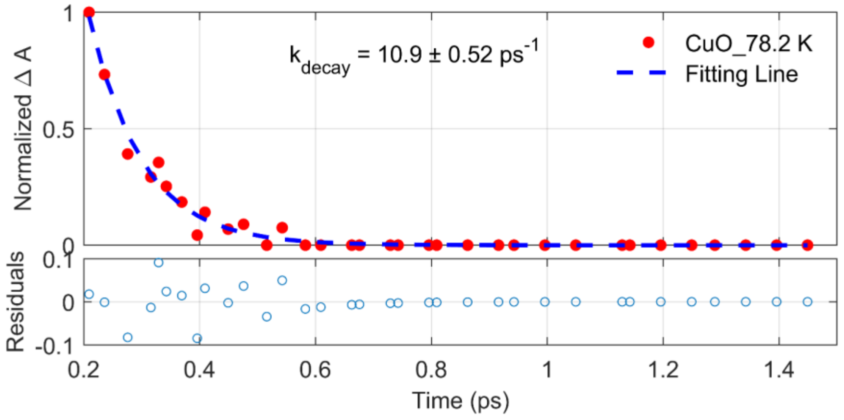

Fig. S83. Top: kinetic analysis of free charge carriers at 78.2 K with a single exponential fitting. The fitted rate constant was inserted in the plot. Bottom: fitting residuals.

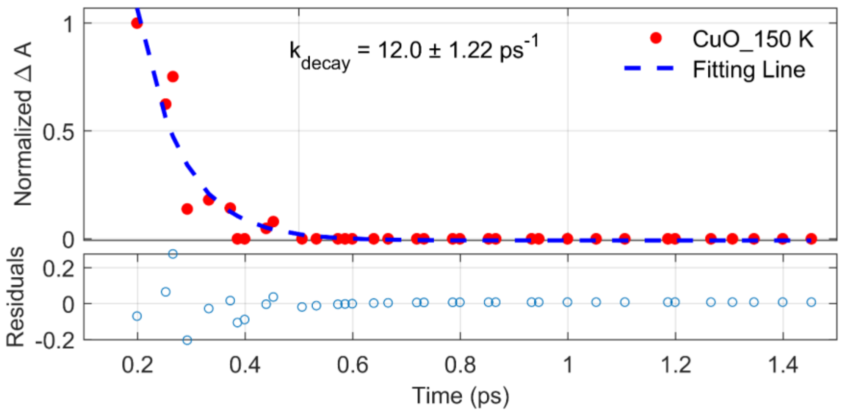

Fig. S84. Top: kinetic analysis of free charge carriers at 150 K with a single exponential fitting. The fitted rate constant was inserted in the plot. Bottom: fitting residuals.

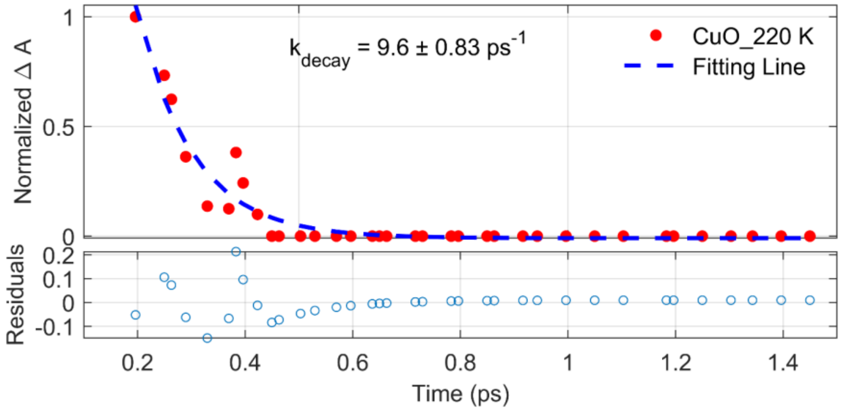

Fig. S85. Top: kinetic analysis of free charge carriers at 220 K with a single exponential fitting. The fitted rate constant was inserted in the plot. Bottom: fitting residuals.

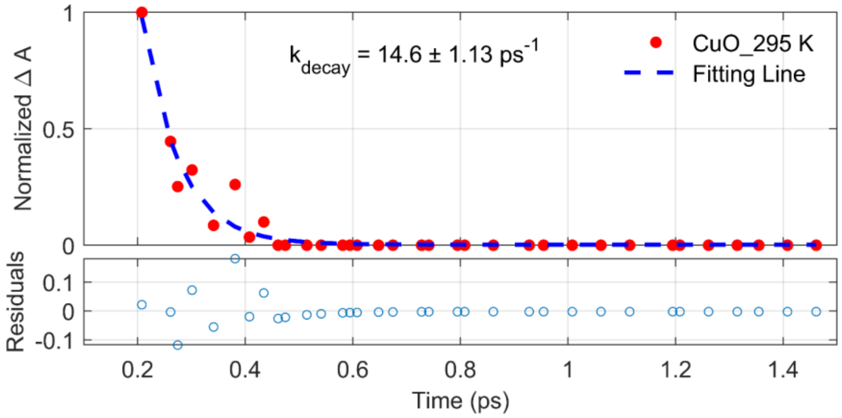

Fig. S86. Top: kinetic analysis of free charge carriers at 295 K with a single exponential fitting. The fitted rate constant was inserted in the plot. Bottom: fitting residuals.

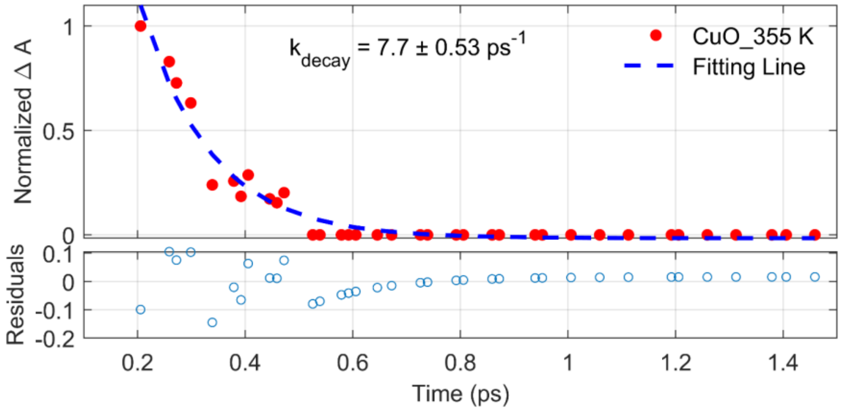

Fig. S87. Top: kinetic analysis of free charge carriers at 355 K with a single exponential fitting. The fitted rate constant was inserted in the plot. Bottom: fitting residuals.

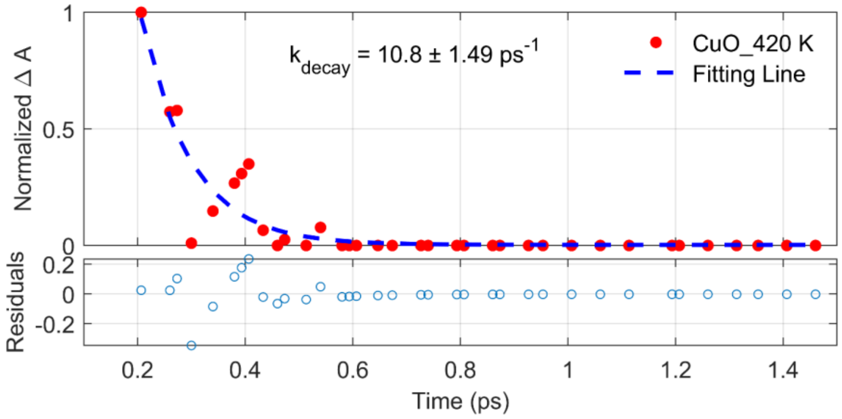

Fig. S88. Top: kinetic analysis of free charge carriers at 420 K with a single exponential fitting. The fitted rate constant was inserted in the plot. Bottom: fitting residuals.

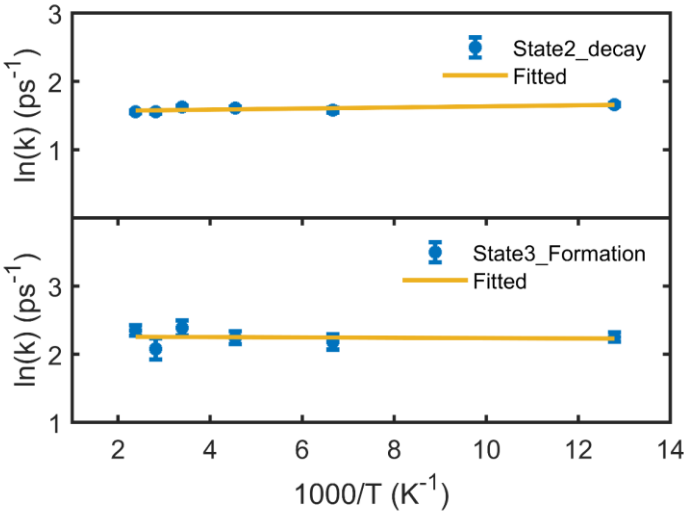

Fig. S89. Temperature dependence of the decay of states2 and formation state3 in Fe_2O_3 film, excited with 3.5 eV (355 nm, fluence of 2.5 mJ·cm^{-2}). The rate constant was fitted by using a single-exponential model.

Reference

1 Toma, F. M.; Cooper, J. K.; Kunzelmann, V.; McDowell, M. T.; Yu, J.; Larson, D. M.; Borys, N. J.; Abelyan, C.; Beeman, J. W.; Yu, K. M. Mechanistic insights into chemical and photochemical transformations of bismuth vanadate photoanodes. *Nat. Commun.* **2016**, *7* (1), 12012.

2 Morris, M. R. Barium titanate: photophysics, photocatalysis & the influence of the ferroelectric effect. University of London, 2018.

3 Sachs, M.; Harnett-Caulfield, L.; Pastor, E.; Davies, B.; Sowood, D. J.; Moss, B.; Kafizas, A.; Nelson, J.; Walsh, A.; Durrant, J. R. Ligand field states control photocatalytic efficiency of transition metal oxides. *Nat. Chem.* **2025**, *17*, 1348–1355.

4 Hassan, I. A.; Parkin, I. P.; Nair, S. P.; Carmalt, C. J. Antimicrobial activity of copper and copper (I) oxide thin films deposited via aerosol-assisted CVD. *J. Mater. Chem. A* **2014**, *2* (19), 2855-2860.

5 Vázquez-Vargas, D.; Pizá-Ruiz, P.; Amézaga-Madrid, P. AACVD system and protocol to fabricate CuO and Co_3O_4 nanostructured coatings for application as selective absorbent materials. *MethodsX* **2023**, *10*, 102219.

6 Zhao, S.; Jia, C.; Shen, X.; Li, R.; Oldham, L.; Moss, B.; Tam, B.; Pike, S.; Harrison, N.; Ahmad, E. The aerosol-assisted chemical vapour deposition of Mo-doped $BiVO_4$ photoanodes for solar water splitting: an experimental and computational study. *J. Mater. Chem. A* **2024**, *12* (39), 26645-26666.

7 Sanz, R.; Jaafar, M.; Hernández-Vélez, M.; Asenjo, A.; Vázquez, M.; Jensen, J. Patterning of rutile TiO_2 surface by ion beam lithography through full-solid masks. *Nanotechnol.* **2010**, *21* (23), 235301.

8 Kresse, G.; Furthmüller, J. Efficient Iterative Schemes for Ab initio Total-Energy Calculations Using a Plane-wave Basis Set. *Phys. Rev. B* **1996**, *54* (16), 11169-11186.

9 Kresse, G.; Hafner, J. Ab initio Molecular Dynamics for Open-shell Transition Metals. *Phys. Rev. B* **1993**, *48* (17), 13115-13118.

10 Dudarev, S. L.; Botton, G. A.; Savrasov, S. Y.; Humphreys, C. J.; Sutton, A. P. Electron-energy-loss spectra and the structural stability of nickel oxide: An LSDA+U study. *Phys. Rev. B* **1998**, *57* (3), 1505-1509.

11 Pastor, E.; Park, J.-S.; Steier, L.; Kim, S.; Grätzel, M.; Durrant, J. R.; Walsh, A.; Bakulin, A. A. In situ observation of picosecond polaron self-localisation in α-Fe_2O_3 photoelectrochemical cells. *Nat. Commun.* **2019**, *10* (1), 3962.

12 Pham, T. D.; Deskins, N. A. Efficient Method for Modeling Polarons Using Electronic Structure Methods. *J. Chem. Theory Comput.* **2020**, *16* (8), 5264-5278.

13 Mosquera-Lois, I.; Kavanagh, S. R.; Walsh, A.; Scanlon, D. O. ShakeNBreak: Navigating the defect configurational landscape. *J. Open Source Softw.* **2022**, *7* (80), 4817.

14 Mosquera-Lois, I.; Kavanagh, S. R.; Walsh, A.; Scanlon, D. O. Identifying the ground state structures of point defects in solids. *npj Comput. Mater.* **2023**, *9* (1), 25.

15 Liao, P.; Carter, E. A. Ab initio DFT+ U predictions of tensile properties of iron oxides. *J. Mater. Chem.* **2010**, *20* (32), 6703-6719.

16 Mosey, N. J.; Liao, P.; Carter, E. A. Rotationally invariant ab initio evaluation of Coulomb and exchange parameters for DFT+ U calculations. *J. Chem. Phys.* **2008**, *129* (1), 014103.

17 Quinn, R. K.; Nasby, R.; Baughman, R. Photoassisted electrolysis of water using single crystal α-Fe_2O_3 anodes. *Mater. Res. Bull.* **1976**, *11* (8), 1011-1017.

18 Merchant, P.; Collins, R.; Kershaw, R.; Dwight, K.; Wold, A. The electrical, optical and photoconducting properties of $Fe_{2-x}Cr_xO_3$ ($0 \leqslant x \leqslant 0.47$). *J. Solid State Chem.* **1979**, *27* (3), 307-315.

19 Marusak, L. A.; Messier, R.; White, W. B. Optical absorption spectrum of hematite, α-Fe_2O_3 near IR to UV. *J. Phys. Chem. Solids.* **1980**, *41* (9), 981-984.

20 Krukau, A. V.; Vydrov, O. A.; Izmaylov, A. F.; Scuseria, G. E. Influence of the exchange screening parameter on the performance of screened hybrid functionals. *J. Chem. Phys.* **2006**, *125* (22), 224106.

21 Mariathasan, J.; Hazen, R.; Finger, L. Crystal structure of the high-pressure form of $BiVO_4$. *Phase Transitions: A Multinational Journal* **1986**, *6* (3), 165-173.

22 Baur, W. H.; Khan, A. A. Rutile-type compounds. IV. SiO_2, GeO_2 and a comparison with other rutile-type structures. *Acta Crystallogr. B* **1971**, *27* (11), 2133-2139.

23 Geller, S. Structure of α-Mn_2O_3,$(Mn_{0.983}Fe_{0.017})_2O_3$ and $(Mn0.37Fe0.63)_2O_3$ and relation to magnetic ordering. *Acta Crystallogr. B* **1971**, *27* (4), 821-828.

24 Åsbrink, S.; Norrby, L.-J. A refinement of the crystal structure of copper (II) oxide with a discussion of some exceptional esd's. *Acta Crystallogr. B* **1970**, *26* (1), 8-15.

25 Tian, L.; Tyburski, R.; Wen, C.; Sun, R.; Abdellah, M.; Huang, J.; D'Amario, L.; Boschloo, G.; Hammarström, L.; Tian, H. Understanding the role of surface states on mesoporous NiO films. *J. Am. Chem. Soc.* **2020**, *142* (43), 18668-18678.

26 Austin, I.; Clay, B.; Turner, C. Optical absorption of small polarons in semiconducting NiO and CoO in the near and far infra-red. *J. Phys. C: Solid State Phys.* **1968**, *1* (5), 1418.
